# Distinct and Dynamic Transcriptome Adaptations of iPSC-Generated Astrocytes after Cytokine Stimulation

**DOI:** 10.3390/cells11172644

**Published:** 2022-08-25

**Authors:** Anna-Sophie Spreng, Markus Brüll, Heidrun Leisner, Ilinca Suciu, Marcel Leist

**Affiliations:** 1 In Vitro Toxicology and Biomedicine, Dept Inaugurated by the Doerenkamp-Zbinden Foundation, University of Konstanz, 78457 Konstanz, Germany; 2 Konstanz Research School Chemical Biology (KoRS-CB), University of Konstanz, 78457 Konstanz, Germany; 3 CAAT-Europe, University of Konstanz, 78457 Konstanz, Germany

**Keywords:** astrocytes, iPSC, inflammation, cytokines, dynamic transcriptome changes

## Abstract

Astrocytes (ACs) do not only play a role in normal neurogenesis and brain homeostasis, but also in inflammatory and neurodevelopmental disorders. We studied here the different patterns of inflammatory activation triggered by cytokines in human induced pluripotent stem cell (iPSC)-derived ACs. An optimized differentiation protocol provided non-inflamed ACs. These cells reacted to TNFα with a rapid translocation of NFκB, while AC precursors showed little response. Transcriptome changes were quantified at seven time points (2–72 h) after stimulation with TNFα, IFNγ or TNFα *plus* IFNγ. TNFα triggered a strong response within 2 h. It peaked from 12–24 h and reverted towards the ground state after 72 h. Activation by IFNγ was also rapid, but the response pattern differed from that of TNFα. For instance, several chemokines up-regulated by TNFα were not affected by IFNγ. Instead, MHC-II-related antigen presentation was drastically enhanced. The combination of the two cytokines led to a stronger and more persistent response. For instance, TRIB3 up-regulation by the combination of TNFα *plus* IFNγ may have slowed NFκB inactivation. Additionally, highly synergistic regulation was observed for inflammation modifiers, such as CASP4, and for STAT1-controlled genes. The combination of the cytokines also increased oxidative stress markers (e.g., CHAC1), led to phenotypic changes in ACs and triggered markers related to cell death. In summary, these data demonstrate that there is a large bandwidth of pro-inflammatory AC states, and that single markers are not suitable to describe AC activation or their modulation in disease, development and therapy.

## 1. Introduction

Upon inflammatory activation, astrocytes (ACs) can adopt new phenotypes and functions, which are different from their normal homeostatic and housekeeping roles in brain physiology [1,2]. Inflammation of ACs may be triggered during infections, by toxicants or in neurodegenerative diseases [3]. Typical AC stimuli are the microglia-derived cytokines TNFα and IL-1. Moreover, some toll-like receptor (TLR) ligands are well known to trigger inflammatory activation [4,5]. The patterns and dynamics of the transcriptome change in human ACs induced by various inflammogen classes require further exploration. 

Many earlier studies have used cultured rodent ACs to study inflammatory activation. Initially, these cells were obtained from neonatal brains and contained glial subpopulations of varying maturity. Disadvantages were contaminations with microglia and other cells [6,7,8,9,10]. Newer approaches allowed the isolation of ACs directly from adult brains [11]. An alternative strategy was to generate ACs directly from murine stem cells [12]. Human ACs strongly differ in their cellular morphology [13], transcriptome profile [14] and functional properties [15,16] from their rodent counterparts. They are also larger and more abundant in human brains than in other species [17]. For instance, mice engrafted with human ACs were shown to be more intelligent [18]. Due to potential species differences, human ACs are often preferred over rodent ACs for studying brain infection and inflammatory diseases relevant to humans. Before human iPSC-derived ACs became available, some pioneering studies were performed with post mortem material (fetal brain or adult brain). For instance, it was already shown 20 years ago that triggers such as TNFα or IFNγ lead to a differential release of chemokines such as CCL5 [19,20]. Subsequent studies [21,22] focused mainly on TNFα as a stimulus, and limitations of source material prevented studies on the dynamics of the transcriptome response. 

Since human brain material is limited for the isolation of primary cells, stem cell-derived ACs have been considered as an important alternative source. Many protocols have been established to differentiate ACs from human pluripotent stem cells [23]. They commonly involve long differentiation processes (several months). Such lengthy procedures make it more difficult to produce functionally identical cells in the end [24] than in protocols generating murine ACs [12]. IPSC-derived ACs first became available for inflammation studies about ten years ago [25]. It took another five years until protocols became available to specifically produce inflammation-competent ACs (e.g., to study acute IL-1 effects) [26]. In these studies, it was found that iPSC-derived ACs remained in a non-activated ground state in serum-containing media, provided the serum level was kept low (≤1%) [27]. With higher serum concentrations, as used for iPSC-derived AC preparations provided by several commercial suppliers, studies of inflammation yielded inconsistent results. Only refined protocols allowed observation of a stimulus-specific activation of ACs by, e.g., IL-1 and IL-6 [28]. The first whole transcriptome study that investigated cytokine effects (IL-1/TNFα) for at least two time points became available recently [29]. A little later, it became clear that different cocktails of inflammogens produce other phenotypes of reactive ACs [30,31]. Very recently, the availability of large amounts of homogeneous iPSC-derived ACs has been used to compare their resting state to the patterns and response dynamics of two distinct stimuli (synuclein; TNFα) [32].

The combined evidence from AC inflammation studies suggests that there is not only one pro-inflammatory state, sometimes also referred to as A1 state [33]. It rather appears that there are multiple patterns of AC activation by cytokines, and each may show particular dynamic features (type and kinetics of activation and return to baseline). 

While many studies have explored the effects of TNFα as stimulus, clearly less is known on IFNγ, another major cytokine involved in brain infections [34,35,36,37,38,39] and chronic autoimmunity, e.g., multiple sclerosis (MS) [40]. Due to the importance of IFNγ in neurological diseases and its emerging role in psychiatric [41,42,43,44] and neurodevelopmental disorders [45,46,47,48,49], the response of human ACs to this cytokine needs further characterization. Presently, only a small set of data from iPSC-derived ACs has been made available in the context of Parkinson’s disease [50]. Data on the interaction of IFNγ and TNFα are scarce.

We aimed here to provide a full data set on the transcriptome responses triggered by TNFα alone, IFNγ alone or the combination of both cytokines. For this purpose, an AC differentiation protocol [51,52] was refined to yield large lots of cells that could be stored frozen and re-used many times in a highly reproducible state after thawing. The characterization of transcriptome dynamics (four stimuli; up to seven time points; three independent repetitions) served as proof of concept that iPSC-derived ACs are ready for many future applications (pharmacologic studies, toxicity screening) requiring consistent cell lots under multiple experimental conditions.

## 2. Materials and Methods

### 2.1. Differentiation and Culture of iPSC-Derived Astrocytes

The iPSC line Sigma iPSC0028 (Si28) (EPITHELIAL-1, #IPSC0028) was differentiated to neuroepithelial stem cells (NESCs) via embryoid body (EB) formation as previously described [53,54]. For further differentiation, the protocol by Palm et al. [51] was adapted and modified to a defined two-step procedure to generate inflammatory-competent astrocytes. An exact overview of cell numbers for seeding, growth rates, morphological characterization during differentiation and hints for differentiation can be found in Table S1. NESCs were thawed and seeded on day −4 at a density of 50,000 cells/cm^2^ in Matrigel™-coated 6-well plates (both Corning, New York, NY, USA) in astrocyte precursor cell (APC) differentiation medium (N2B27 medium (1): 50% Dulbecco’s modified Eagle’s medium/F12 (DMEM/F12) (Gibco^®^ by Thermo Fisher Scientific, Waltham, MA, USA), 50% neurobasal medium (Gibco^®^ by Thermo Fisher Scientific, Waltham, MA, USA), 2 mM L-glutamine (Sigma-Aldrich, Traufkirchen, Germany), 1 × B27 without vitamin A (Gibco^®^ by Thermo Fisher Scientific, Waltham, MA, USA) and 1 × N2 (Gibco^®^ by Thermo Fisher Scientific, Waltham, MA, USA) supplemented with 150 μM ascorbic acid (Sigma-Aldrich, Traufkirchen, Germany), 3 μM CHIR99021 (Axon MedChem, Groningen, the Netherlands), 0.5 μM purmorphamine (Enzo Life Sciences, Lörrach, Germany) and 20 ng/mL FGF2 (Peprotech, Hamburg, Germany)). Medium was exchanged on day −2 and day −1. On day 0, cells were re-seeded at a density of 40,000 cells/cm^2^ in astrocyte progenitor cell (APC) maintenance medium (N2B27 medium (2): Dulbecco’s modified Eagle’s medium/F12 (DMEM/F12) 2 mM l-glutamine, 1 × B27 with vitamin A, and 1 × N2 supplemented with 40 ng/mL FGF2, 40 ng/mL EGF (Peprotech, Hamburg, Germany) and 0.15 µL/mL (*v*/*v*) hLIF (Merck, Darmstadt, Germany)). APCs were split every 3–4 days (40,000 cells/cm^2^ for splitting after 3 days or 20,000 cells/cm^2^ for splitting after 4 days) and medium was exchanged every other day. After 2 weeks, APCs can be frozen. 

Differentiation to astrocytes was achieved by seeding APCs on Matrigel™-coated 6-well plates at a density of 40,000 cells/cm^2^ in APC maintenance medium. Medium was exchanged every other day. After 4 days, APC maintenance medium was replaced with astrocyte differentiation medium (N2B27 medium (2): Dulbecco’s modified Eagle’s medium/F12 (DMEM/F12) 2 mM l-glutamine, 1 × B27 with vitamin A, and 1 × N2 supplemented with 1% fetal bovine serum (FBS) (PAA Laboratories, Cölbe, Germany)). Astrocytes were split once a week and seeded at a density of 100,000 cells/cm^2^ in Matrigel™-coated 10 cm dishes. After 5 weeks of differentiation from APCs, astrocytes were frozen.

### 2.2. Re-Seeding of APCs and Astrocytes

Cells were detached by aspiration of the medium and incubation with Accutase (PanBiotech, Aidenbach, Germany) for 5–8 min at 37 °C, 5% CO_2_. Cells were completely washed off with DMEM/F12, cell suspension was transferred into 50 mL Falcon conical tubes (Corning, New York, NY, USA) and centrifuged at 500× *g* for 5 min. Supernatant was removed and cell pellet was re-suspended in respective media. Cells were counted using a Neubauer counting chamber (VWR, Radnor, PA, USA) and cells were seeded in indicated cell numbers and plate formats in respective media for further experiments.

### 2.3. Freezing of APCs and Astrocytes

APCs and astrocytes were frozen in their respective culture medium with 10% dimethyl sulfoxide (DMSO, Merck, Darmstadt, Germany). Cells were detached by aspiration of the medium and incubation with Accutase for 5–8 min at 37 °C, 5% CO_2_. Cells were completely washed off with DMEM/F-12, cell suspension was transferred into 50 mL Falcon tubes (Corning, New York, NY, USA) and centrifuged at 500× *g* for 5 min. Supernatant was removed and cell pellet was re-suspended in chilled respective freezing mix and transferred into cryopreservation vials (Sarstedt, Nümbrecht, Germany). After storage at −80 °C in a freezing container (Thermo Scientific™ Mr. Frosty™) for 1 day, cells were transferred and stored in N_2liq_.

### 2.4. Coating of Cell Culture Plates

For maintenance culture of NESCs and differentiation of APCs and astrocytes, 6-well plates were coated with Matrigel™ (1:40 in DMEM/F-12) and incubated for 30 min at 37 °C and 5% CO_2_. Afterwards, unadsorbed Matrigel™ was aspirated and cells were directly seeded onto plates. 

For NFκB translocation quantification, Western blot and transcriptome sample generation, 96-well plates were coated with 43 μg/mL poly-L-ornithine hydrobromide (PLO, Sigma-Aldrich, St. Louis, MO, USA) and 1 μg/mL fibronectin (Sigma-Aldrich, St. Louis, MO, USA) in Milli-Q H_2_O. After incubation at 37 °C overnight, plates were washed three times with Milli-Q H_2_O, dried and stored at 4 °C until further use. 

For immunofluorescence staining of cells, cover slips with a diameter of 13 mm (Thermo Fisher Scientific, Waltham, MA, USA) were transferred into 24-well plates and coated with Matrigel™ 1:20 in DMEM/F-12 at 37 °C and 5% CO_2_ for 30 min.

### 2.5. RNA Extraction, cDNA Synthesis and Real-Time qPCR

Extraction of total RNA was performed using the TRIzol reagent (PeqGOLD Trifast™ (VWR, Radnor, PA, USA), according to the manufacturer’s instructions. One microgram of total RNA was reverse transcribed with i-Script™ Reverse Transcription Supermix (Bio-Rad™, Hercules, CA, USA) and quantitative real-time PCR was performed with SsoFast™ EvaGreen^®^ Supermix (Bio-Rad™, Hercules, CA, USA) using a CFX96 Real-Time PCR Detection System (Bio-Rad™, Hercules, CA, USA) with the Bio-Rad CFX Manager Software v2.0 (Bio-Rad™, Hercules, CA, USA) for determination of the cycle threshold values. The primer sequences used are summarized in Table S6. Results were analyzed using the ΔΔCt method with initial normalization to PGK1 and RPL13A and depicted as fold change relative to the gene expression in NESCs.

### 2.6. Immunofluorescence Staining

Cells were seeded either on Matrigel™-coated glass cover slips (Thermo Fisher Scientific, Waltham, MA, USA) or 96-well plates at a density of 50,000 cells/cm^2^ in respective media. After 3 days, cells were fixed by replacing half the medium with 10% neutral buffered formalin (Leica Biosystems Richmond, Inc., Richmond, IL, USA) and incubated for 30 min at room temperature. Fixation solution was removed and cells were washed once by addition of Dulbecco’s phosphate buffered saline (DPBS) w/o Ca^2+^, Mg^2+^ (Gibco^®^ by Thermo Fisher Scientific, Waltham, MA, USA). Cells were permeabilized in 0.6% Triton^®^-X100 Sigma-Aldrich, St. Louis, MO, USA) in DPBS for 10 min at room temperature. Afterwards, cells were blocked for 1 h by replacing permeabilization buffer with 0.1% Triton^®^-X100 and 5% FBS in DPBS (blocking buffer) at room temperature. Incubation with the respective primary antibodies at indicated dilutions in blocking buffer (Table S7) was performed at 4 °C overnight. Non-bound primary antibodies were removed and cells were washed three times with DPBS. Then, incubation with respective secondary antibodies (Table S7), diluted in blocking buffer, was performed for 1 h at room temperature. Nuclei were counterstained by the addition of Hoechst-33342 (H-33342; Merck, Darmstadt, Germany) together with the secondary antibodies. After washing 3 times in DPBS, cells in 96-well plates were stored in DPBS at 4 °C until further use and cells grown on glass cover slips were mounted using Aqua-Poly/Mount (Polyscience, Warrington, PA, USA) face down onto object slides, left to dry overnight at room temperature and then stored at 4 °C until further use.

To quantify immunopositive ACs, three independent AC differentiations were used for imaging and three fields per differentiation were scored. Fields were randomly picked in the H-33342 channel (about 70 cells/field) and the number of immunopositive cells was determined by the overlay with the channel of interest.

Cells stained with immunofluorescent antibodies were imaged using a Zeiss AxioObserver epifluorescence microscope with ZEN 2 pro blue edition software (Zeiss, Oberkochen, Germany) and images were processed in ImageJ (FIJI version 1.51s). 

### 2.7. Quantification of Morphological Changes

For quantification of astrocyte morphology after inflammatory stimulation, at least 4 images per condition with about 100 cells/field were analyzed. To this end, astrocytes were cultivated on glass coverslips for 2 days before they were stimulated for 72 h with inflammatory cytokines or a solvent control. Cells were fixed and immunostained against GFAP. Nuclei were counterstained with H-33342. Images were taken with a Zeiss AxioObserver with a 63× Plan-Apochromat oil objective.

Images were analyzed with FIJI (version 1.53f51). Images were background corrected with a rolling ball algorithm. The gamma value was set to 0.7. The minimum pixel value was increased until cell-free areas appeared as black. To obtain binary images, images were then auto-thresholded (method = mean). Binary images of the GFAP channel were iteratively reduced to lines with FIJI’s built-in “skeletonize” function. Skeletons were pruned to avoid overestimation of branching of cells without distinct processes. The skeletonized images were analyzed with the built-in “analyze skeleton” function. To obtain the number of cells per field, binary images of the H-33342 channel were analyzed. Particles >10 µm² were counted as nuclei. Overlapping nuclei were separated by a “watershed” function. The total length of branches and the number of branches in each field was normalized to the number of nuclei of the respective field. An overview of the used workflow can be found in Figure S16.

### 2.8. NFκB Translocation

Cells were seeded on fibronectin/poly-L-ornithine-coated 96-well plates at a density of 50,000 cells/cm^2^ in respective medium. After 3 days, cells were stimulated by the addition of TNFα (final concentration of 10 ng/mL), IFNγ (final concentration of 20 ng/mL), TNFα (final concentration of 10 ng/mL) plus IFNγ (final concentration of 10 ng/mL) or medium only as control and incubated at 37 °C and 5% CO_2_ for 30 min. Afterwards, cells were fixed by replacing half the medium with 10% neutral buffered formalin (Leica Biosystems Richmond, Inc, Richmond, IL, USA) and incubated for 30 min at room temperature. Fixation solution was removed and cells were stored in DPBS at 4 °C. Immunofluorescent staining was performed as described using anti-NFκB p65 antibody (Table S7) and nuclei were counterstained with Hoechst-33342. 

The translocation of the nuclear factor B (NFκB) p65 subunit was quantified using a Cellomics ArrayScan™ automated microscope with the predefined algorithm “nuclear translocation” as described previously [5,55]. In brief, the outline of the nuclei in the H-33342 channel (λ_ex_ = 365 nm) was determined first. From there, the mean average pixel intensities of the nuclear area (5 pixels; 3.3 µm away from the nuclear outline) and the mean average pixel intensities of a ring in the cytoplasm (width of 4 pixels; 2.6 µm and distance to the nuclear outline of 5 pixels; 3.3 µm) were measured. The ratio of the average pixel intensities of the nuclear area and the cytoplasmic area were calculated. “Activated cells” were defined when the ratio exceeded the average ratio of unstimulated reference cells by at least one SD. The average intensity ratio of the reference wells was automatically obtained. For analysis, three independent astrocyte differentiations were used and images were taken from 3 technical replicate wells each. In each well, 300 cells were automatically used for analysis.

### 2.9. Western Blot

For Western blot analysis of proteins, 500,000 astrocytes were seeded into each well of a 6-well plate (Corning, New York, NY, USA) in astrocyte medium. After 3 days, 20 µM ruxolitinib (Selleckchem, Munich, Germany) was added to some wells. After 30 min, 10 ng/mL TNFα or 20 ng/mL IFNγ (both from R&D Systems, Mineapolis, MN, USA) were added and incubated for 1 h. Then, cells were lysed in 120 µL 1× Laemmli buffer and heated for 5 min at 95 °C. Lysates were centrifuged for 1 min at 10,000× *g* through NucleoSpin Filters (Macherey-Nagel GmbH, Düren, Germany) to break down long DNA strands. Twenty microliters of lysates were loaded onto 10% SDS gels and gels were run for 30 min at 80 V and then at 120 V until bands reached the bottom of the gel. Proteins were transferred onto nitrocellulose membranes (Amersham, Buckinghamshire, UK) using the iBlot™ 2 dry blotting system (Invitrogen, Waltham, MA, USA). Membranes were blocked with 5% BSA (*w*/*v*) in TBS-Tween (0.1% (*v*/*v*)) for 1 h. Respective primary antibodies were incubated at 4 °C overnight (Table S8). After washing three times with 5% BSA (*w*/*v*) in TBS-Tween (0.1% (*v*/*v*)) at room temperature for 10 min each, membranes were incubated with horseradish peroxidase-conjugated secondary antibodies for 1 h at RT (Table S8). For visualization, ECL Western blotting substrate (Pierce/Thermo Fisher Scientific, Rockford, IL, USA) was used and imaged with a Fusion-SL 3500 WL device with Fusion software (Bio-Rad™, Hercules, CA, USA). Antibodies used for Western blot analysis are specified in Table S8.

### 2.10. Treatment of Astrocytes for Transcriptome Sample Generation

Astrocytes were seeded at a density of 50,000 cells/cm^2^ in 96-well plates. After 24 h, cells were treated with 10 ng/mL TNFα, 20 ng/mL IFNγ, the combination of TNFα (10 ng/mL) plus IFNγ (20 ng/mL) or 100 ng/mL lipopolysaccharide (LPS) from Escherichia coli O55:B5 (Merck, Darmstadt, Germany) in a reverse manner and cells were incubated for 72 h, 24 h, 18 h, 12 h, 8 h, 6 h, 4 h or 2 h with the cytokines or with medium as solvent control.

### 2.11. Transcriptome Data Generation and Analysis

For preparation of astrocyte, APC and NESC transcriptome samples, 50,000 cells/cm² were seeded in 96-well plates. After respective treatments, sample lysates were prepared as described previously [53,56]. Briefly, medium was aspirated and cells were lysed in 33 µL of 1× enhanced BioSpyder lysis buffer (BioSpyder Tech., Glasgow, UK), incubated for 10 min at 37 °C and stored at −80 °C before shipment on dry ice. Samples were prepared from two independent biological replicates with two technical replicates each. 

Measurements were performed at Bioclavis (BioSpyder Tech., Glasgow, UK) via the TempO-Seq targeted sequencing technology [57] applied to the EuTox 2.0 gene subset (4041 probes for 3562 unique genes, detailed information can be found in Supplementary Workbook). The resulting FASTQ files were aligned using the STAR algorithm to a pseudo-transcriptome by BioClavis to receive a raw count table.

The data were analyzed by employing the R package DESeq2 (v1.24.0) [58]. The DESeq2 object was constructed from raw counts of mRNA species and normalized with DESeq2′s median of ratios normalization for differential gene expression analysis. For marker expression analysis, counts were normalized to total counts per sample (counts per million, CPM). A Wald test was used for the statistical analysis of differential gene expression in the treatment group against the untreated control group. In the case of differentiation analysis, gene expression was analyzed relative to NESCs. For a gene to be considered differentially expressed, the threshold of Benjamini–Hochberg adjusted *p*-values (p_adj_) was set to ≤0.05. An absolute log2 fold change threshold relative to the control group of ≥0.5 was additionally set.

GO term and transcription factor (Transcriptional Regulatory Relationships Unraveled by Sentence-based Text mining, TRRUST) analysis was performed using Metascape’s multiple gene list function with express analysis [59] *(*https://metascape.org/, accessed on 11 February 2022). Venn diagrams were calculated and drawn using the online tool at https://bioinformatics.psb.ugent.be/webtools/Venn/ (accessed on 6 March 2022).

### 2.12. Statistics

Experiments were performed on at least three independent cell preparations, with several technical replicates for each batch, if not otherwise stated. Information concerning descriptive statistics and experimental variability is included in the figure legends or the figures themselves. For significance testing and data display, GraphPad Prism 5 software (Version 7.04, GraphPad Software, Inc., San Diego, CA, USA) was used.

## 3. Results

### 3.1. Two-Step Differentiation of Astrocytes from Neural Stem Cells

**Figure 1:**
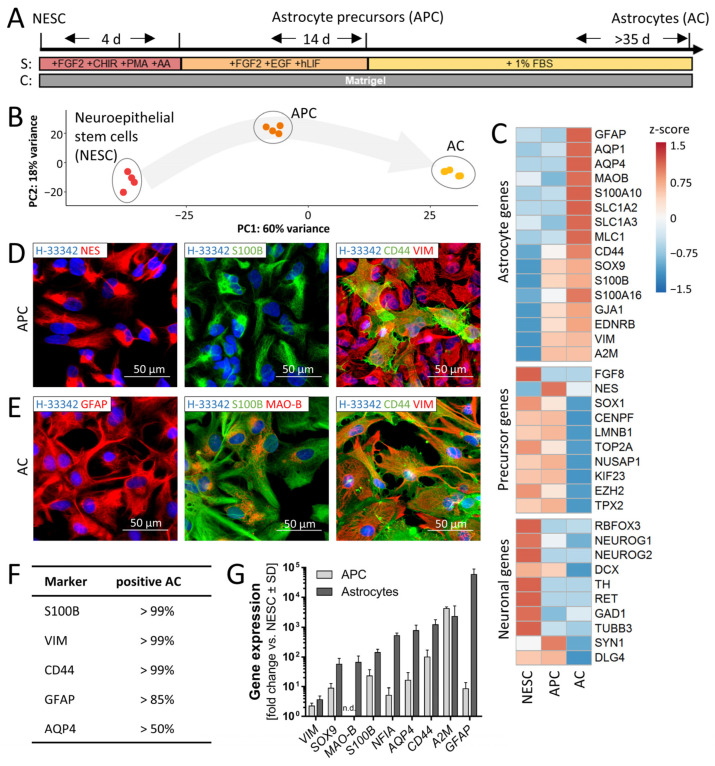
Generation and characterization of human astrocytes. (**A**) Schematic differentiation of astrocytes (ACs) from neuroepithelial precursor cells (NESCs) via astrocyte precursor cells (APCs). NESCs were thawed and differentiated to APCs in two steps. For the first four days, the medium contained fibroblast growth factor (FGF2), the Wnt activator (CHIR99021), the smoothened receptor agonist purmorphamine (PMA) for activation of the hedgehog pathway and the antioxidant ascorbic acid (AA) as supplements (S). In the second phase (14 d), the medium was supplemented with FGF2, epidermal growth factor (EGF) and human leukemia inhibitory factor (hLIF). APCs were differentiated towards ACs with 1% fetal bovine serum (FBS) as medium supplement. ACs were considered mature after at least 35 days of differentiation from APCs. (**B**) The gene expression of NESCs, APCs and ACs was quantified for a panel of 3562 genes (four samples each). In the 2-dimentional display of the principal component analysis (PCA), the axes are scaled according to the variance explained by the respective PCA component. (**C**) Heat map depicting the row-wise z-scores of expression levels of selected marker genes in NESCs, APCs and ACs. Data are derived from mean expression levels (in counts per million reads) with *n* = 4. (**D**) APCs were fixed and immunostained for typical astrocyte precursor markers: nestin (NES), S100B, CD44 and vimentin (VIM). Nuclei were counterstained with the DNA stain Hoechst-33342 (H-33342). (**E**) Mature ACs were fixed and immunostained for typical astrocyte markers: GFAP, S100B, monoamine oxidase B (MAO-B), CD44 and vimentin (VIM). Nuclei were stained with H-33342 (blue). Representative epifluorescence images of typical expression patterns and morphologies are shown in (**D**,**E**). Pseudocoloring of antigens is indicated in image legends. (**F**) Quantification of the percentage of marker-positive ACs after ≥35d. Three independent AC differentiations were used for imaging and three fields per differentiation were scored. Cells (70/field) were randomly picked in the H-33342. (**G**) AC marker expression was confirmed by RT-qPCR gene expression analysis in APCs (14 d) and ACs (35 d). Data are means ± SD of ≥3 independent differentiations. Gene expression data (RT-qPCR) for later time points of AC differentiation can be found in Figure S1A. n.d., not detected.

To generate inflammation-competent astrocytes, we optimized a previously established protocol [51,52]. As the starting point of the two-step procedure, we chose neuroepithelial stem cells (NESCs). These can be generated in large amounts from pluripotent stem cells [53,54] and are suitable as a source population over many passages (P3–P10). NESCs were differentiated in the first step to astrocyte precursor cells (APCs) within 14 days. The second differentiation step was usually continued directly from there to generate mature astrocytes (ACs) (Figure 1A). Alternatively, APCs could be amplified for some days or be frozen, before they were used to generate ACs. The expression of AC markers (GFAP, CD44, AQP4, S100B, MAO-B) increased from day 1 (=APC) to 35 (=AC) of this differentiation stage. After the second step (day 35), ACs remained stable for at least 90 days. It was possible to freeze them on day 35 and to use them for further experiments after thawing without loss of markers (Figure S1A, Table S1). 

The progress of differentiation was characterized by a targeted transcriptome analysis (panel of 3562 genes) [53]. A principal component analysis (PCA) of the gene expression patterns of NESCs, APCs and ACs showed a clear separation of the cell populations on the transcriptome level, and a high reproducibility across differentiation runs (Figure 1B). According to their profile of stage-specific markers [14,30], ACs generated here faithfully replicated the expression pattern found in human brains (Figure 1C and Figure S1B). For instance, the expression of fetal or immature astrocyte markers, such as NUSAP1 or TOP2A [14], was enhanced in APCs but low in ACs. 

To characterize the intermediate and final cell populations on a single cell level, we performed immunostaining (Figure 1D,E). The majority of APCs were positive for nestin, S100B and vimentin. A minor population of APCs was also already positive for the astrocyte-restricted precursor marker CD44 (Figure 1D). All cells in AC cultures stained for CD44. Additionally, mature ACs were also positive for glial fibrillary acidic protein (GFAP) (>85%) and aquaporin 4 (AQP4) (>50%) (Figure 1 E,F). A minor subpopulation of monoamine oxidase B (MAO-B)-positive cells was also detected (Figure 1E). These mature AC markers were completely absent in APCs (not shown). 

In order to confirm the transcriptome data, we performed RT-qPCR analysis on a set of established AC genes [12,14,30]. Data from this test confirmed that early-stage astrocyte markers (SOX9, S100B, CD44) were already strongly expressed in APCs relative to their NESC precursor state. The expression of these genes was then further increased in ACs. PCR data also confirmed that mature astrocyte markers (MAO-B, GFAP) were strongly up-regulated in AC (Figure 1G)

In summary, this phenotyping approach showed that the two-stage differentiation strategy successfully generated ACs from a multipotent, highly proliferative precursor population (NESCs). High AC purity was obtained despite the fact that NESCs are naturally biased towards neuronal differentiation (high RBFOX3, NEUROG1, NEUROG2, TUBB3 expression). The initial protocol step converted NESCs to APCs, an intermediate population with a distinct gliogenic marker profile (SOX9, S100B, vimentin). This allowed the generation of pure AC cultures in the second step. The cell population obtained thereby did not contain any neurons, showed negligible amounts of immature precursors and had nearly arrested proliferation (Table S1).

### 3.2. Inflammatory Competence of iPSC-Derived Astrocytes after Stimulation with Cytokines

**Figure 2:**
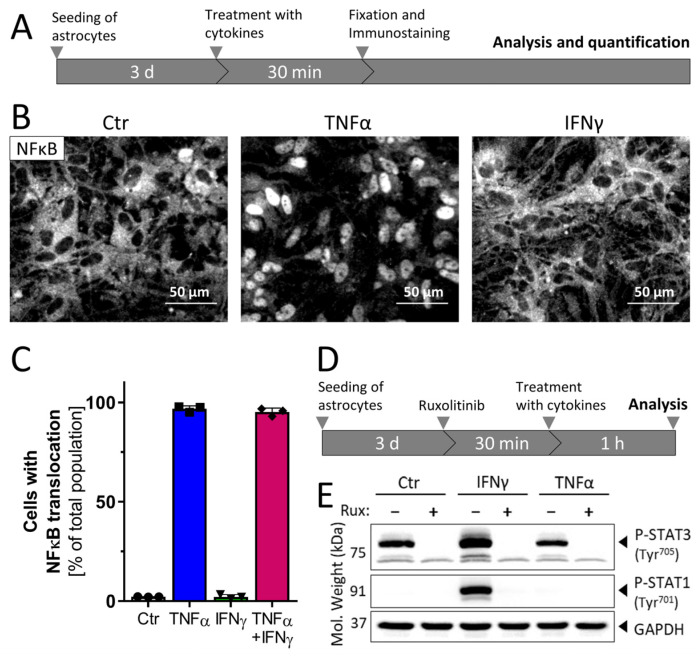
Signal transduction in astrocytes after stimulation with cytokines. (**A**) Schematic representation of the experimental protocol used for determination of NFκB translocation in ACs. Three days after seeding (50,000 cells/cm^2^), cells were stimulated with cytokines: TNFα (10 ng/mL), IFNγ (20 ng/mL) or the combination of TNFα (10 ng/mL) *plus* IFNγ (20 ng/mL). At 30 min after treatment, ACs were fixed and immunostained against the NFκB subunit p65. (**B**) Representative immunofluorescent images showing the NFκB p65 signal in control, TNFα- or IFNγ-stimulated astrocytes. The nuclear counterstain (Hoechst-33342) is not shown here but is illustrated in Figure S2. (**C**) Quantification of NFκB translocation was performed using an ArrayScan™ high-content imaging device. Data are shown as the percentage of cells with a nuclear NFκB p65 localization. Data are means of three independent astrocyte differentiations ± SD. (**D**) Schematic representation of the experimental protocol used for Western blot analysis. ACs were pre-treated with ruxolitinib (20 µM) for 30 min, and then stimulated for 1 h with TNFα (10 ng/mL) or IFNγ (20 ng/mL) before sampling for Western blot analysis. (**E**) Astrocyte lysates were analyzed using anti-phophoSTAT1 (Tyr^701^), anti-phosphoSTAT3 (Tyr^705^) and anti-GAPDH antibodies. Controls to verify equal amounts of STAT1/3 were not run, as increases in protein amounts were not expected within the short duration of the experiments. The displayed blot is representative of three experiments.

The activation of ACs by many important cytokines converges on the NFκB pathway [60]. We investigated this signaling event, in order to ascertain the inflammatory competence of ACs. Two typical, but biologically distinct, pro-inflammatory cytokines (TNFα and/or IFNγ) were used as stimuli, and the fractions of cells showing NFκB p65 translocation were quantified (Figure 2A). Control cells showed a strong immunoreactivity in the cytoplasm and no signal in the nuclei. Treatment with TNFα induced a translocation of NFκB p65 into the nuclei within 30 min (Figure 2B and Figure S2). ACs treated with IFNγ showed a staining pattern similar to non-stimulated controls (Figure 2B and Figure S2). None of the treatments affected cell viability, even if this was followed for up to 72 h.

The quantification of the fraction of ACs with a predominantly nuclear p65 localization revealed that almost every cell exposed to TNFα showed NFκB translocation. Cells treated with IFNγ showed no translocation, but cells exposed to TNFα plus IFNγ were all activated (Figure 2C). The observed response pattern was specific to ACs, as NESCs and APCs showed only a minor response to TNFα (Figure S3). Thus, this assay demonstrated the functional maturation (inflammatory competence) of ACs; the source population used to generate them was non-responsive (<5 % reactivity). Moreover, these findings indicate that the iPSC-derived astrocytes are a homogeneous population in terms of inflammatory reactivity to TNFα.

IFNγ is an inflammogen known to signal fundamentally differently than TNFα, by activating the JAK-STAT pathway [60,61]. To confirm the role of this type of signal transduction in ACs, the phosphorylation of STAT (P-STAT1 and P-STAT3) was investigated (Figure 2D). Western blot analysis showed that STAT3 was phosphorylated (P-STAT3) in control ACs (Figure 2E). This was expected, since P-STAT3 is important for the expression of astrocytic genes during development [62]. Treatment with the JAK inhibitor ruxolitinib completely blocked P-STAT3 formation, in line with a constitutively active branch of the JAK-STAT3 pathway in ACs. Phosphorylated STAT1 (P-STAT1) was not observed in control ACs. Stimulation with IFNγ led to a phosphorylation of this transcription factor (P-STAT1), which was completely blocked by ruxolitinib. Thus, IFNγ activated the JAK-STAT1 axis in ACs, similar to its role in several other cell types [61].

All together, these experiments show that the iPSC-derived mature ACs react to diverse inflammatory stimuli in specific ways consistent with literature knowledge [61,63]. At least two clearly different pathways were utilized, and it was therefore interesting to investigate whether the diverse signaling responses were reflected by distinct activation patterns.

### 3.3. Distinct Patterns of Gene Expression after Exposure to Three Types of Inflammogens

**Figure 3:**
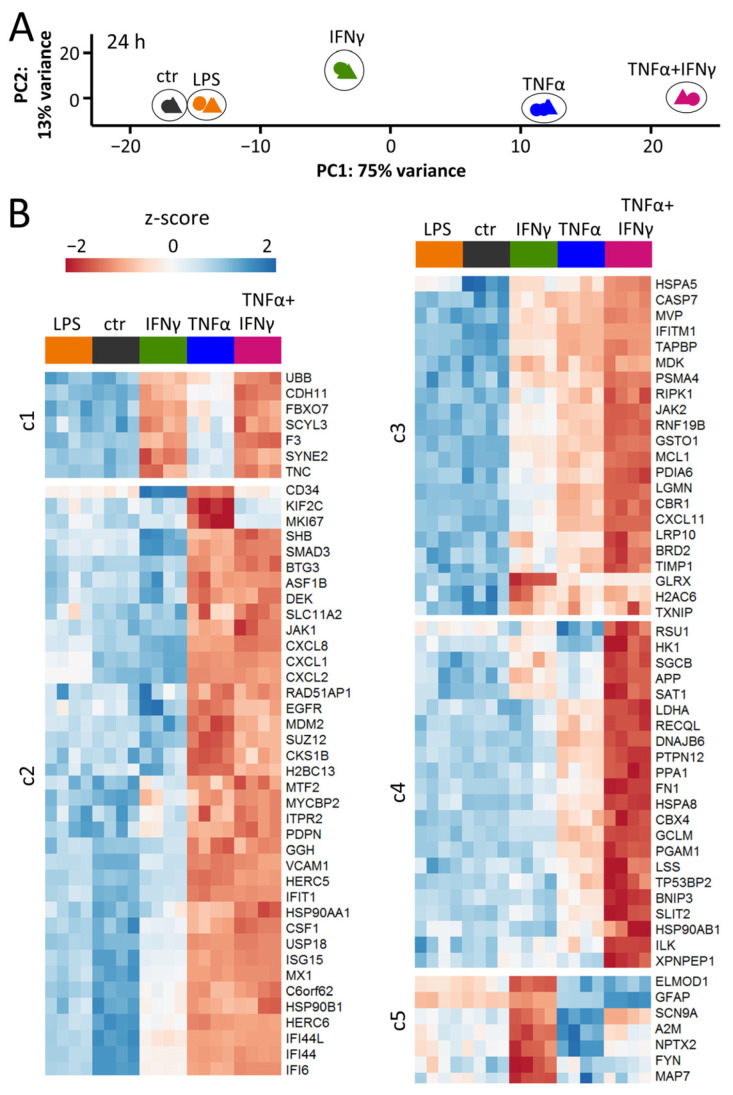
Distinct patterns of gene expression after exposure to three types of inflammogens. ACs were seeded at a density of 50,000 cells/cm^2^ into 96-well plates. Three days after seeding, cells were stimulated with TNFα (10 ng/mL), IFNγ (20 ng/mL), a combination of TNFα (10 ng/mL) *plus* IFNγ (20 ng/mL) or lipopolysaccharide (LPS; 100 ng/mL). After 24 h, cells were lysed and gene expression profiling was performed for 3562 genes using RNASeq analysis (TempOSeq method). (**A**) Principal component analysis (PCA) representing the different responses of astrocytes to LPS, IFNγ, TNFα and co-treatment with TNFα *plus* IFNγ. Ctr = untreated ACs. The axes are scaled according to the variance covered. Note that four samples for each condition are displayed, but some are not visible due to the overlap of data points. (**B**) For each of the four stimuli, the top 30 differentially expressed genes (DEGs based on fold change) were selected. This pool of 96 transcripts is displayed as a heat map of row-wise z-scores. The heat map blocks were obtained by non-supervised clustering. Clusters (c) 1–5 represent genes regulated by: IFNγ (c1), TNFα (c2), IFNγ and TNFα (c3), co-treatment with TNFα *plus* IFNγ (c4), IFNγ alone but not in combination with TNFα (c5). The rows represent the four samples used for each condition.

To obtain a comprehensive overview of the AC activation responses, we used transcriptome analysis. For this purpose, cells were stimulated with TNFα, IFNγ or TNFα plus IFNγ for 2, 4, 8, 12, 18, 24 and 72 h. Additionally, ACs were exposed to the gram-negative bacterial endotoxin lipopolysaccharide (LPS). While detailed time-course data are discussed below, we first concentrated on the response patterns and their differences after 24 h of stimulation. A PCA of the gene expression patterns of ACs after this exposure time showed a highly reproducible response under all conditions. All cytokine-driven activation states separated clearly from the control and from each other (Figure 3A). 

The transcriptome pattern after LPS stimulation was slightly different from control ACs. This suggests that the cells hardly reacted to the bacterial-derived inflammogen. A more detailed view (volcano plot) showed that only 13 genes were up-regulated (to a moderate extent) at 24 h and an additional analysis at 8 h (to avoid missing transient regulations) showed even less effect (Figure S4). These findings are consistent with the few reports on LPS effects in human AC cultures. They are fully in line with demonstrations that pure murine ACs do not react to LPS, unless they are pre-activated [5,6,7]. This confirms a role of ACs as the second line of defense against pathogens after invaders have been recognized by microglia [4].

ACs stimulated with IFNγ (24 h) up-regulated 118 genes (Figures S5 and S6). TNFα triggered a wider transcriptome response (230 DEGs up-regulated) (Figure 3B, Figures S5 and S7). The strongest response was found when ACs were stimulated with the combination of TNFα plus IFNγ (Figure S8). Amongst the 366 up-regulated genes, 131 were unique to this treatment (Figure S5). Non-supervised clustering of the transcriptome responses at 24 h indicated five major reaction patterns: (c1) genes regulated by IFNγ (e.g., thromboplastin (F3)); (c2) genes regulated by TNFα (e.g., the signaling factor SHB); (c3) genes regulated by both IFNγ and TNFα (e.g., caspase 7); (c4) genes regulated synergistically by TNFα plus IFNγ (e.g., GCLM); (c5) genes regulated by IFNγ alone but not in combination with TNFα (e.g., alpha-macroglobulin (A2M)) (Figure 3B). 

The transcriptome data were also examined by bioinformatics tools to identify potential upstream transcriptional regulators responsible for the different responses. The typical RFX transcription factors involved in the regulation of MHC-II genes [64,65] were specifically over-represented for IFNγ-induced genes. JUN, REL and NFΚBIA were only found to be enriched for TNFα. Transcription factor binding sites for, e.g., NFΚB or RELA, were found to be over-represented amongst the promoter regions of all cytokine-induced gene sets (Figure S9).

We were also interested in how our data relate to other AC inflammation studies. A particular combination of inflammogens, TNFα, IL1α and C1q (termed TIC), has often been used to generate and characterize neurotoxic ACs [30,66]. A recent study on TIC-induced reactive ACs revealed a distinct signature of six cell-surface markers: VCAM-1, BST2, ICOSL, HLA-E, PD-L1 and PDPN after 24 h of stimulation [31]. Our transcriptome analysis contained probes for three of these markers (VCAM-1, HLA-E and PDPN) which were all up-regulated by TNFα at 24 h (IFNγ failed to regulate PDPN). The response in our system was characterized by the expression of genes involved in immune effector recruitment and activation, similar to the findings by Labib et al. [31]. The study on TIC-activated ACs identified proteomics-based activation markers. Altogether, 24 of the most regulated markers (Figure 4D in [31]) were detectable by our transcriptome approach. In ACs stimulated with TNFα or TNFα plus IFNγ, we found 23 of them to be up-regulated (Table S2). Thus, TNFα-activated ACs in our study show many similarities to TIC-stimulated neurotoxic ACs.

Overall, these findings show that not only were the signaling pathways triggered by cytokines in ACs different and distinct, but the downstream responses were also unique. Having shown the suitability of the iPSC-derived AC culture model to study inflammatory patterns, we moved on to characterize the full response dynamics.

### 3.4. Rapid Cytokine-Induced Gene Expression Changes in ACs

**Figure 4:**
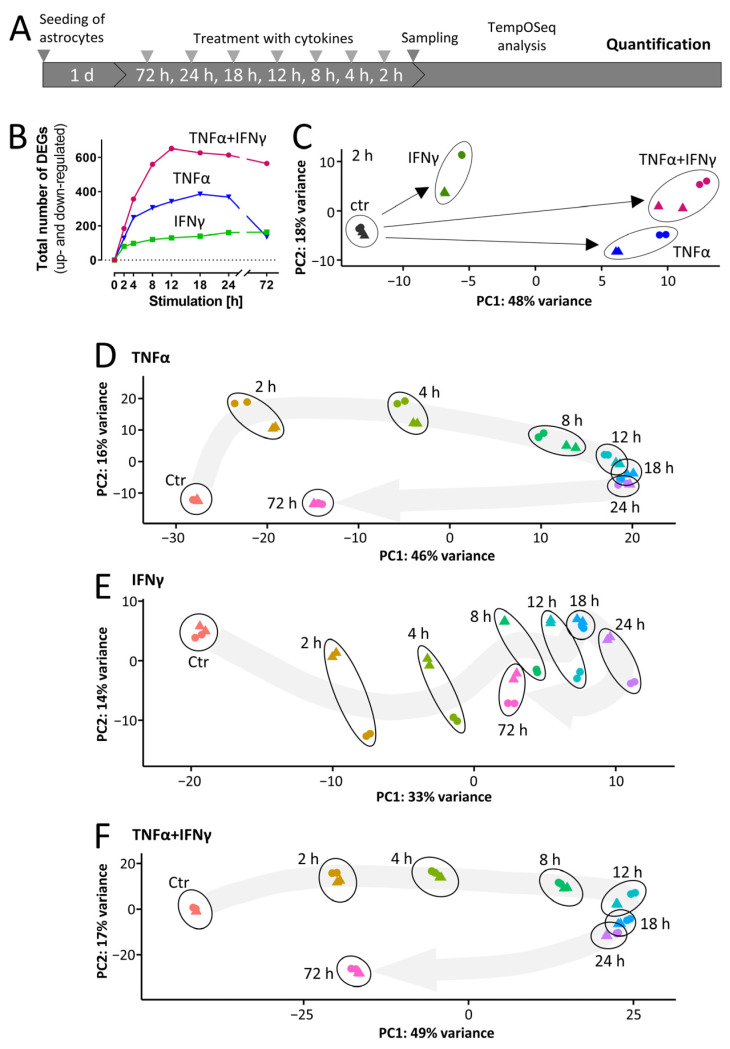
Dynamics of cytokine-induced gene expression in ACs. (**A**) Schematic representation of the experimental procedure for AC sample generation and targeted RNASeq analysis (TempOSeq method). ACs were seeded at a density of 50,000 cells/cm^2^ into 96-well plates. On the following days, cells were stimulated with TNFα (10 ng/mL), IFNγ (20 ng/mL) or a combination of TNFα (10 ng/mL) *plus* IFNγ (20 ng/mL). After the indicated time periods, cells were lysed and gene expression profiling was performed for 3562 genes. (**B**) The total numbers of differentially expressed genes (DEGs) are depicted. Genes were considered as differentially expressed with *p*-value ≤ 0.05 (after false discovery rate adjustment) and an absolute log_2_fold change of ≥0.5. A full list of all genes and their regulation can be found in Supplementary Materials. (**C**) Principal component analysis (PCA) of the early transcriptome responses (2 h) to TNFα, IFNγ and co-treatment of TNFα *plus* IFNγ. Triangles and circles show samples belonging to one differentiation batch. The PCA axes are scaled according to the variance covered. (**D**–**F**) The time-dependent changes in gene expression in astrocytes in response to (**D**) TNFα, (**E**) IFNγ and (**F**) co-treatment with TNFα *plus* IFNγ were analyzed by principal component analysis (PCA). The first two PCA dimensions are shown with the axes scaled according to the variance covered. Triangles/circles indicate samples belonging to one differentiation batch. The gray arrows represent the overall trend of gene regulation, they are fitted to the average PCA position of the four samples.

To obtain information on inflammatory responses over time, ACs were treated for up to 72 h with TNFα, IFNγ or the combination of the two. Transcriptome analysis was performed on eight time points including the control (Figure 4A). The kinetics of regulation showed different patterns (Figure 4B). While all treatments induced significant transcriptome responses after 2 h, these clearly differed from each other (Figure 4C and Figure S10A,B). IFNγ up-regulated 67 genes (Figure S10A,B) and, e.g., IRF1 was identified as one of the likely upstream regulators (Figure S10C). TNFα triggered the up-regulation of 95 genes (*n* = 145, if together with IFNγ) and NFκB was identified as one of the most likely early upstream regulators (Figure S10C). The group of genes (*n* = 16) up-regulated early by all cytokines contained IRF1, CCL2, PTX3 and NRG1. These four general inflammation markers may be useful to identify activated ACs in other in vitro or in vivo studies.

### 3.5. Overall Dynamics of Transcriptome Changes

To gain insights into the dynamics of the responses to the cytokines, PCA maps were assembled. These are 2D representations of the overall transcriptome changes for each cytokine over time (Figure 4D–F). For instance, TNFα induced markedly different response features at 2, 4 and 8 h. Further changes became smaller and were minimal from 12–24 h. Then, a large step was evident for the 72 h time point, when the overall transcriptome change on the PCA map and the number of DEGs had mostly returned to control level (Figure 4B,D and Figure S11A,B). The extent of the regulation of key genes and the evidence for the role of transcription factors in their regulation also tended to decrease at 72 h. However, the NFκB family transcription factors appeared to play a dominant role from 2 h until 72 h (Figure S11C).

The increase in total DEGs in IFNγ-treated ACs was 2-fold lower than for TNFα and showed a continuous increase over time (Figure 4B and Figure S12A,B). For the transcriptional regulators related to antigen presentation (e.g., RFX5, RFXAP, CIITA), it seemed to take 8–12 h until they affected the transcriptome (Figure S12C). The PCA map did not show a strong return towards control level at 72 h (Figure 4E). 

Concerning the number of DEGs, the transcriptome responses of IFNγ and TNFα were additive (Figure 4B and Figure S13). The PCA map indicated that a maximum of regulation occurred from 12–24 h, with a partial return towards control level at 72 h (Figure 4F).

Our data on the transcriptome dynamics suggest that ACs can adopt a large variety of inflammatory states, depending on the type of inflammogen and on the duration of stimulation. The model system established here may be used to delineate further types of activation states. This may eventually help to define markers for various acute or chronic disease states or for treatment success of anti-inflammatory drugs. Moreover, it would be interesting to better understand whether different activation states are also reflected by, e.g., a change in metabolism or altered interactions with surrounding cells. Here, we studied the regulation dynamics of individual genes more closely to provide a better basis for the definition of inflammation markers.

### 3.6. Differential Regulation of Exemplary Genes Coding for Inflammation Markers

**Figure 5:**
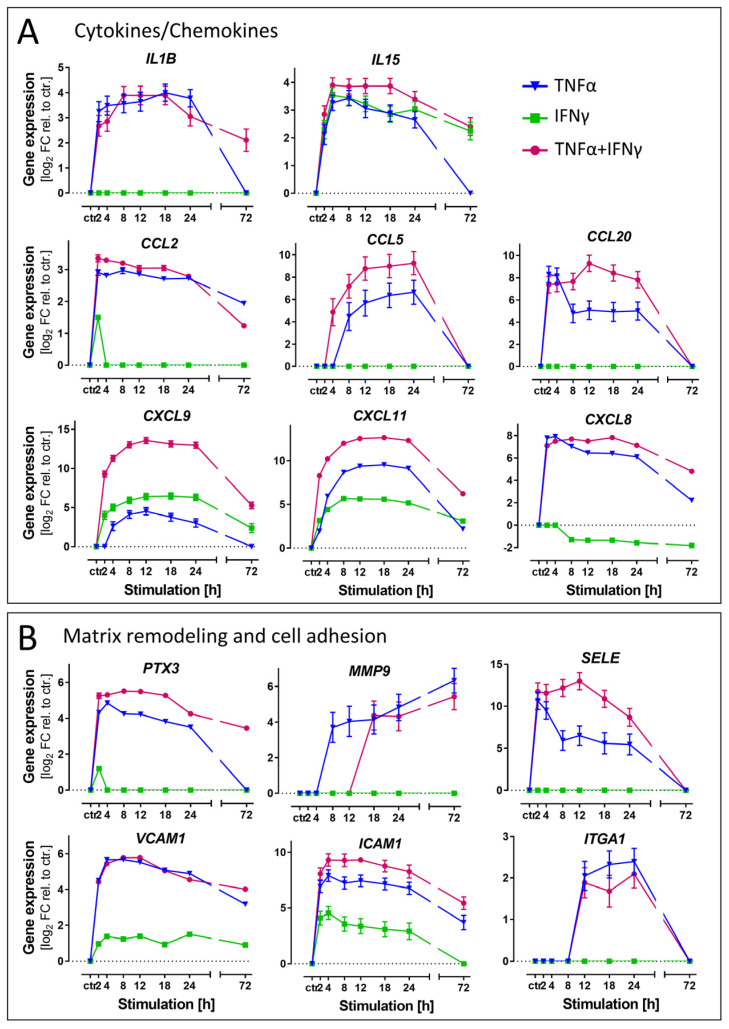
Differential regulation of exemplary genes coding for inflammation markers. Gene expression data were obtained from ACs as detailed in Figure 4. (**A**) Levels of mRNA for exemplary cytokines/chemokines. (**B**) Levels of mRNA for exemplary proteins involved in matrix remodeling and cell adhesion. All data are means ± SD from four samples (two samples each from 2 independent differentiations). Ctr.: unstimulated control ACs.

GO term analysis pointed clearly to a role of cytokine-stimulated ACs in chemotaxis and immune cell activation. This biological domain was chosen to assess differential gene regulations triggered by TNFα vs. IFNγ. We selected chemokine/cytokine genes, which are related to the invasion of immune effector cells into the CNS (Figure 5 and Figure S14). For instance, IL15, CXCL9 and CXCL11 were up-regulated in all treatment conditions. Some other chemokines (e.g., CCL2, CCL5, CCL20) were only up-regulated by TNFα (not by IFNγ) (Figure 5A).

A second, but different, function of ACs is the regulation of endothelial blood–brain barrier (BBB) integrity in a context-dependent manner [67]. We selected representative genes involved in matrix remodeling and cell adhesion [68,69]. For pentraxin 3 (PTX3), matrix metallopeptidase 9 (MMP9), E-selectin (SELE) or an integrin component (ITGA1), we observed a strong (>20 fold) enhancement of transcription by TNFα, but no effect of IFNγ. Cell adhesion molecules (VCAM1, ICAM1) facilitating astrocyte–leukocyte interactions were up-regulated in all three conditions, but the response to IFNγ was always the weakest (Figure 5B).

From these results, we conclude that ACs exposed to different cytokines show distinct behaviors with respect to immune cell invasion into the brain. TNFα is most likely a main driver involved in the invasion of leukocytes, while IFNγ may be involved in other mechanisms.

### 3.7. Differential Effects of Cytokines on Genes Related to Antigen Presentation

**Figure 6:**
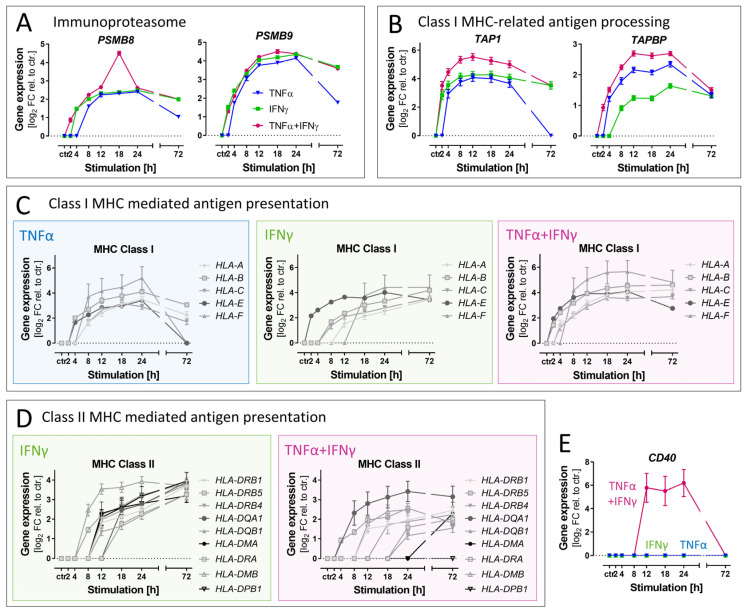
Differential effects of cytokines on the antigen presentation program in astrocytes. Gene expression data were obtained from ACs as detailed in Figure 4. Genes involved in antigen processing and presentation were selected for detailed analysis. (**A**) Levels of mRNA for immunoproteasome subunits. (**B**) Levels of mRNA for proteins involved in the transport of peptides to be presented on MHC class I. (**C**) Levels of mRNA for MHC class I proteins. (**D**) Levels of mRNA for MHC in class II proteins. Note that TNFα effects are not displayed as this cytokine did not affect the expression of MHC class II genes. (**E**) Exemplification of cytokine synergy by CD40, a pivotal enhancer on antigen-presenting cells.

There is rising evidence that not only microglia, but also ACs, can act as antigen-presenting cells under neuroinflammatory conditions [70]. Therefore, we investigated the expression changes in genes related to this function. Genes encoding catalytic subunits (PSMB8, PSMB9) of the immunoproteasome [40] were strongly up-regulated in all treatment conditions (Figure 6A). The same was true for several genes necessary for class I MHC-related antigen processing and presentation (Figure 6B,C). IFNγ is known for its special role in professional antigen-presenting cells, concerning the expression of MHC class II molecules. Cultured astrocytes from rodents and humans do not constitutively express MHC class II. However, they may change their phenotype upon IFNγ treatment [70,71,72,73]. Therefore, the up-regulation of genes encoding MHC class II molecules was examined in ACs generated here (Figure 6D). Indeed, IFNγ triggered a broad up-regulation. TNFα alone did not enhance the transcription of MHC class II genes at any tested time point and even delayed the expression triggered by IFNγ. Our bioinformatics analysis showed that upstream transcriptional regulators related to MHC class II molecules (RFXAP, RFX5) were identified only for the IFNγ-containing treatment conditions (Figure S12).

Finally, our analysis also identified a gene (CD40) that was regulated >50-fold by the co-stimulation with TNFα plus IFNγ, but not by any of the cytokines alone (Figure 6E). The role of CD40 in inflamed human astrocytes is poorly characterized, and it needs to be tested whether the gene expression data presented here translate into functional proteins.

Our analysis, related to a potential role of ACs in antigen presentation, revealed that some genes are targeted by IFNγ, but not by the classical inflammogen TNFα. The example of CD40 showed that at least three distinct inflammatory response patterns can be distinguished even with the small panel of cytokines used here.

### 3.8. Characterization of Response Features of ACs after Prolonged Cytokine Exposure

**Figure 7:**
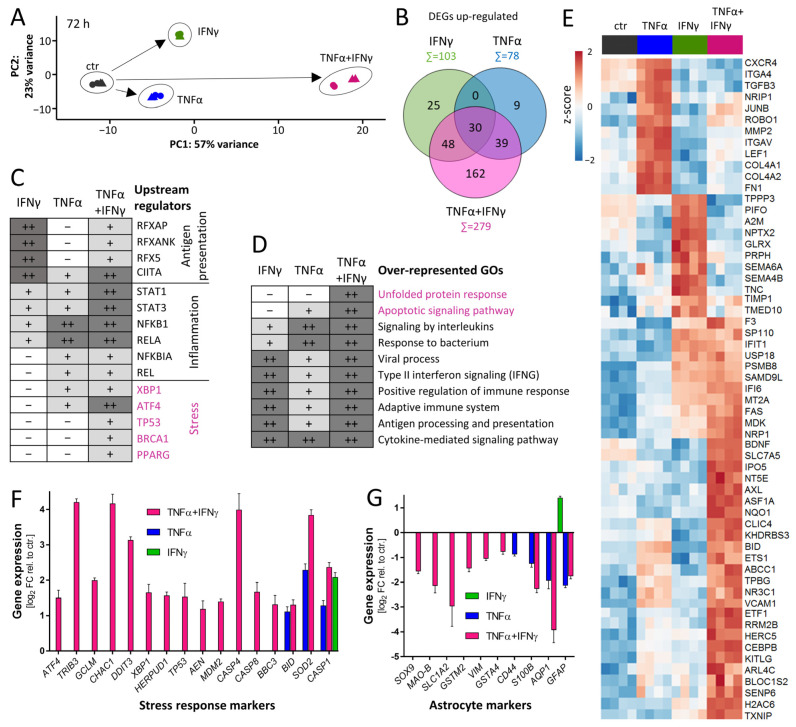
Characterization of response features of ACs after prolonged cytokine exposure. AC transcriptome data were obtained as detailed in Figure 4. Data analysis was focused on the 72 h time point. (**A**) Principal component analysis (PCA) with arrows indicating the direction and extent of cytokine-induced transcriptome changes. The axes are scaled according to the variance covered. (**B**) Venn diagram depicting the number of genes up-regulated in ACs ≥ 2-fold and with padj < 0.05. Numbers in overlapping areas represent the genes commonly regulated. Gene names can be found in Table S3. (**C**) Transcription factors whose binding sites were over-represented on promoters of up-regulated DEGs were identified [59] and labeled here as “upstream regulators”. The boxes of the table indicate the extent to which a stimulus regulated genes controlled by the indicated transcription factors. Dark gray (++) represents strong over-representation (*p* < 10^−12^), light gray (+) represents moderate over-representation (10^−12^ < *p* < 0.05) and white (-) represents no significant over-representation. Major biological roles of the transcription factors are indicated to the right. Full set of data can be found in Table S4. (**D**) Gene ontology (GO) analysis was performed [59] with genes induced ≥ 2-fold (padj < 0.05) in ACs stimulated for 72 h. Ten of the top 20 over-represented GOs are shown. Significance is coded as in (**C**). Full set of data can be found in Table S5. (**E**) For each treatment condition, the top 20 differentially expressed genes (DEGs based on fold change) were selected. A heat map (row-wise z-scores) was assembled for this pool of 59 DEGs. (**F**) Regulation of genes encoding proteins involved in stress response pathways in ACs stimulated for 72 h with IFNγ, TNFα or the combination of TNFα plus IFNγ. (**G**) Regulation of AC marker genes. Data are means ± SD of 4 samples.

After the general characterization of the dynamics of the transcriptional changes and the examination of exemplary gene groups, we were interested in the adaptions of ACs to prolonged exposure (72 h). A PCA showed that the groups related to the different cytokine treatments were clearly separated and that the co-treatment with TNFα plus IFNγ induced the most extensive transcriptome changes (Figure 7A and Figure S15). The number of up-regulated DEGs at 72 h was higher for IFNγ (*n* = 103) than for TNFα (*n* = 78) (Figure 7B). The co-treatment with the two cytokines affected 162 genes in a highly synergistic way (Figure 7B, Table S3). This indicated that co-treatment for prolonged times might lead to a response pattern, which is clearly distinct from that caused by any single cytokine. 

The prediction of the upstream regulators by the bioinformatics algorithm revealed a unique group of stress-related transcription factors in the co-treated ACs (ATF4, XBP1, TP53) (Figure 7C, Table S4). Additionally, gene ontology over-representation analysis confirmed the findings that stress response genes, annotated to the GOs “unfolded protein response” and “apoptotic signaling pathway”, were strongly up-regulated (Figure 7D, Table S5). Although the viability controls run within the experimental time period (up to 72 h) did not indicate a significant cell loss, it is possible that ACs being continuously stimulated for even longer periods would eventually die. Indeed, cell death (apoptosis or pyroptosis) has been repeatedly described for continuously stimulated glia [74]. To visualize the response pattern of some individual transcripts, the top 20 DEGs of each treatment condition were selected and pooled. Their representation in a clustered heat map gives an overview of prominent inflammatory genes after prolonged AC stimulation. One example of a strongly synergistic gene, that was not easily predicted beforehand, is the receptor tyrosine kinase AXL (Figure 7E). By additionally using GO over-representation analysis, we identified stress-response marker genes that were synergistically regulated (Figure 7F). They contained caspase 8 (CASP8), the transcription factor p53 (TP53) and ER-stress genes (DDIT3, GCLM, ATF4), all of which have been related to cell death processes. Additionally, we found a strong down-regulation of typical astrocyte marker genes in ACs treated with the combination of TNFα plus IFNγ for 72 h (Figure 7G). This may be interpreted as signs for de-differentiation under stressful conditions. 

We conclude that prolonged exposure of ACs to the combination of the pro-inflammatory cytokines TNFα plus IFNγ may not only trigger a unique inflammatory phenotype, but also may affect the viability and functionality of the cells.

### 3.9. Morphological Changes in Astrocytes Triggered by Inflammatory Stimuli

**Figure 8:**
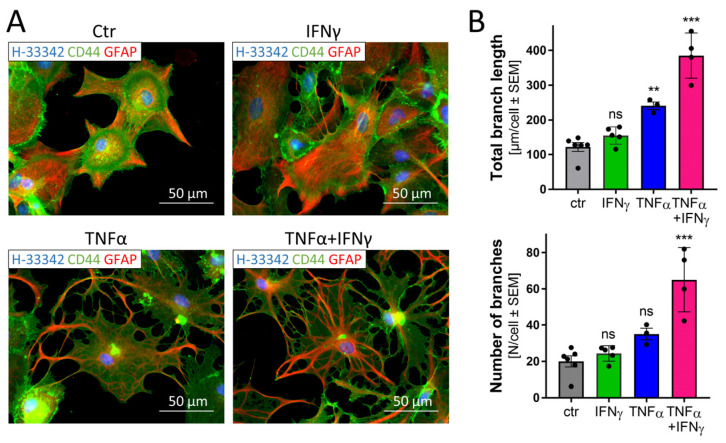
Morphological changes in astrocytes triggered by inflammatory stimuli. ACs (50,000 cells/cm^2^) were seeded on Matrigel™-coated glass cover slips in 24-well plates. After stimulation for 72 h with TNFα (10 ng/mL), IFNγ (20 ng/mL) or the combination of TNFα plus IFNγ, cells were fixed and stained with anti-CD44 and anti-GFAP antibodies and visualized using an epifluorescence microscope (63× oil objective). Nuclei were counterstained with Hoechst-33342. (**A**) Representative images. (**B**) The AC morphology was analyzed using an algorithm determining branch length and the number of branches per cell. Three to five fields (black data points) with an average of about 100 cells per condition were analyzed. Ctr.: untreated ACs. ** *p* < 0.01; *** *p* < 0.001 according to ANOVA with Dunnett’s post hoc test. ns, not significant.

Reactive ACs are known to change their morphology in response to injury or under pathological conditions [75]. As the transcriptome analysis at late time points (72 h) suggested a stressed cell state that may well lead to morphological changes, we examined the cellular phenotype. Cells exposed to TNFα, IFNγ or TNFα plus IFNγ were immunostained and imaged (Figure 8A).

IFNγ did not induce any obvious morphological changes in the ACs stained for GFAP and CD44. The combined treatment with TNFα plus IFNγ induced severe morphological changes, while TNFα alone triggered mild changes. To obtain an unbiased measure of cell morphology, we used an algorithm that quantified total branch length and the number of branches of ACs. The cytokine co-treatment led to significant alterations in this endpoint (Figure 8B and Figure S16).

This analysis confirmed that the cytokines not only change the AC transcriptome profile but also lead to morphological changes similar to those observed in primary cultures or human brains [75,76,77,78].

## 4. Discussion

Human iPSC-derived ACs, produced by a two-step protocol, reacted to diverse inflammatory stimuli in similar ways as has been described for primary cells. The high yield and consistency of our AC cultures allowed the recording of dynamic transcriptome changes that were specific for the tested inflammogens. Clearly distinct inflammatory reactions were observed here for various stimuli and for different times following stimulation. These in vitro findings suggest that there might also be a large diversity of glial responses to cytokines, e.g., upon infection, following birth complications (infection; hypoxia) or during neurodegenerative processes. Our findings suggest that NFκB-activating cytokines, such as TNFα, would be mainly responsible for the immediate chemotactic signals that recruit leukocyte infiltration into the brain. In disease situations, where IFNγ takes a dominant role, the response dynamics would be slower, and an important feature would be the shift from an innate to an adaptive immune response (antigen presentation to T cells). Our data also suggest that strong inflammatory activation of ACs (e.g., by cytokine combinations) may not only change their function, but also their morphology and, possibly, even their viability. 

For macrophages and microglia, it has become common to distinguish two fundamentally different types of activation by cytokines, the so-called M1/M2 states [79,80]. There have been suggestions that astrocytes may also take on a pro-inflammatory A1 state and an anti-inflammatory A2 state. However, there has also been some debate on whether such a simple classification scheme adequately describes the reaction pattern of glial cells [81,82,83,84]. Our data suggest that even with a single stimulus, cells can be found in largely distinct activation states, depending on the time period of exposure. Moreover, two cytokines triggered largely differing pro-inflammatory states (e.g., after 24 h), and the combination of the two cytokines, TNFα *plus* IFNγ, even triggered a third distinct state, characterized by the up-regulation of several genes not regulated by the individual cytokines. It is thus likely that there is a large bandwidth of inflammation states. These may play different roles in brain development, neuronal plasticity or in infectious and degenerative diseases [85,86]. The study of the consequences of various states of inflammatory activation for metabolic functions, cell–cell interactions and survival has to date been hampered by the limited availability of suitable cells. More such studies should now be possible with model systems as the one described here. 

On the basis of a better availability of high numbers of cells, the full spectrum of AC responses after stimulation with various inflammogens can be mapped. Potential stimuli to be used for such research may not only be cytokines, but also microbial/viral danger signals, complement components and proteins/cell constituents linked to neurodegenerative disease. For instance, in murine ACs, ligands of the TLR2 receptor have been found to be more important than TLR4 ligands (such as LPS) [5] and it will be important to explore this for iPSC-derived human ACs. Disease-associated synuclein has already been established as an inflammogen [32], while the situations for amyloid-beta aggregates, prions and TDP-43 and tau-aggregates need further investigation. This is relevant, as there is evidence for a role of ACs in various neurodegenerative diseases [87] and in particular in Alzheimer’s disease [31].

The culture system presented here may be particularly useful for such studies, as the two-step procedure could be used to generate different AC phenotypes. It has been shown earlier that the generation of a sharply defined precursor population is key to the generation of murine ACs from iPSCs [12]. We found here that the APCs resemble such a precursor population (expression of typical gene set). It should thus be tried in the future to use APCs as starting point for different AC populations by exchanging FBS for other differentiation factors or cocktails thereof.

Another possibility may be to expose ACs themselves to differentiation factors. We found that FBS could be removed from the cultures after 35 days of differentiation. Under such conditions, and at high cell densities, ACs generated here had virtually no cell cycle activity. They could, e.g., be combined with neurons and a constant number maintained over at least one week [52]. This feature would allow the study of factors that change the overall phenotype (without inflammatory activation) or that trigger AC proliferation.

The morphological changes and the activation of stress-response pathways after prolonged and strong stimulation suggest that ACs would change their overall function. It will be interesting to find out in which way they alter their neuronal support properties, whether they may eventually undergo apoptosis/pyroptosis (or another type of cell death) and how such processes may be affected by toxicants or infection. For rodent ACs, the possibility to return to a stem cell state, to convert to neurons or to contribute to neurorepair processes has been suggested [88,89,90]. This is less clear for human ACs, and cultures with inflammatory competence, dynamic activation patterns and a quiescent ground state, as described here, should help to answer such questions.

## Data Availability

Additional raw data can be requested from the corresponding author.

## Supplementary Materials

The following supporting information can be downloaded at: https://www.mdpi.com/article/10.3390/cells11172644/s1, Figure S1: Gene expression of human astrocytes during differentiation, Figure S2: Translocation of NFκB in astrocytes after stimulation with cytokines, Figure S3: NFκB translocation in AC precursor populations after stimulation with cytokines, Figure S4: Transcriptional response of ACs to stimulation with LPS, Figure S5: Genes uniquely expressed in ACs stimulated with cytokines for 24 h, Figure S6: Transcriptional response of ACs to IFNγ stimulation for 24 h, Figure S7: Transcriptional response of ACs to TNFα stimulation for 24 h, Figure S8: Transcriptional response of ACs to combined treatment with TNFα *plus* IFNγ for 24 h, Figure S9: Overrepresentation of transcription factor binding sites amongst ACs regulated by cytokines, Figure S10: Response of AC to cytokines upon stimulation for 2 h, figure S11: Time-dependent transcriptome response of ACs to TNFα stimulation, Figure S12: Time-dependent transcriptome response of ACs to IFNγ stimulation, Figure S13: Time-dependent transcriptome response of ACs to combined TNFα *plus* IFNγ stimulation, Figure S14: Differential regulation of additional genes coding for inflammation markers, Figure S15: Response of ACs to cytokines upon stimulation for 72 h, Figure S16: Schematic workflow for the quantification of morphological changes in ACs stimulated with cytokines, Table S1: Additional information on the differentiation, Table S2: Comparison of markers with TIC-induced astrocytes, Table S3: Overview of genes uniquely differentially expressed (DEGs) in ACs stimulated with cytokines for 72 h, Table S4: Overview and exact significance numbers of top 20 deregulated transcription factors (72 h), Table S5: Overview and exact significance numbers of top 20 enriched gene sets (72 h), Table S6: Overview of primers used for real-time qPCR, Table S7: Antibodies used for immunofluorescent staining, Table S8: Antibodies used for western blot. Raw and analyzed transcriptome data can be found in “Supplementary workbook.xlsx”.
