# Supplementary material for: Distinct and Dynamic Transcriptome Adaptations of iPSC-Generated Astrocytes after Cytokine Stimulation
Source: Cells. 2022 Aug 25;11(17):2644. doi: 10.3390/cells11172644 (PMC9455058; doi:10.3390/cells11172644)
Supplement: Supplementary file 1 [file cells-11-02644-s001.zip › Spreng et al., 2022, Supplementary file.pdf]

Supplementary information for

Distinct and dynamic transcriptome adaptations of iPSC-generated astrocytes after cytokine stimulation

Anna-Sophie Spreng [1,2], Markus Brüll [1], Heidrun Leisner [1], Ilinca Suciu [1,2] and Marcel Leist [1,3,*]

Table of Contents

Figure S1	P2	Gene expression of human astrocytes during differentiation
Figure S2	P3	Translocation of NFκB in astrocytes after stimulation with cytokines
Figure S3	P4	NFκB translocation in AC precursor populations after stimulation with cytokines
Figure S4	P5	Transcriptional response of ACs to stimulation with LPS
Figure S5	P6	Genes uniquely expressed in ACs stimulated with cytokines for 24 h
Figure S6	P7	Transcriptional response of ACs to IFNγ stimulation for 24 h
Figure S7	P8	Transcriptional response of ACs to TNFα stimulation for 24 h
Figure S8	P9	Transcriptional response of ACs to combined treatment with TNFα *plus* IFNγ for 24 h
Figure S9	P10	Overrepresentation of transcription factor binding sites amongst ACs regulated by cytokines
Figure S10	P11	Response of ACs to cytokines upon stimulation for 2 h
Figure S11	P12	Time-dependent transcriptome response of ACs to TNFα stimulation
Figure S12	P13	Time-dependent transcriptome response of ACs to IFNγ stimulation
Figure S13	P14	Time-dependent transcriptome response of ACs to combined TNFα *plus* IFNγ stimulation
Figure S14	P15	Differential regulation of additional genes coding for inflammation markers
Figure S15	P16	Response of ACs to cytokines upon stimulation for 72 h
Figure S16	P17	Schematic workflow for the quantification of morphological changes in ACs stimulated with cytokines
Table S1	P18	Additional information on the differentiation
Table S2	P19	Comparison of markers with TIC-induced astrocytes
Table S3	P20	Overview of genes uniquely differentially expressed (DEGs) in ACs stimulated with cytokines for 72 h
Table S4	P21	Overview and exact significance numbers of top 20 deregulated transcription factors (72 h)
Table S5	P22	Overview and exact significance numbers of top 20 enriched gene sets (72 h)
Table S6	P23	Overview of primers used for real-time qPCR
Table S7	P24	Antibodies used for immunofluorescent staining
Table S8	P25	Antibodies used for western blot
	P26	Supplementary references

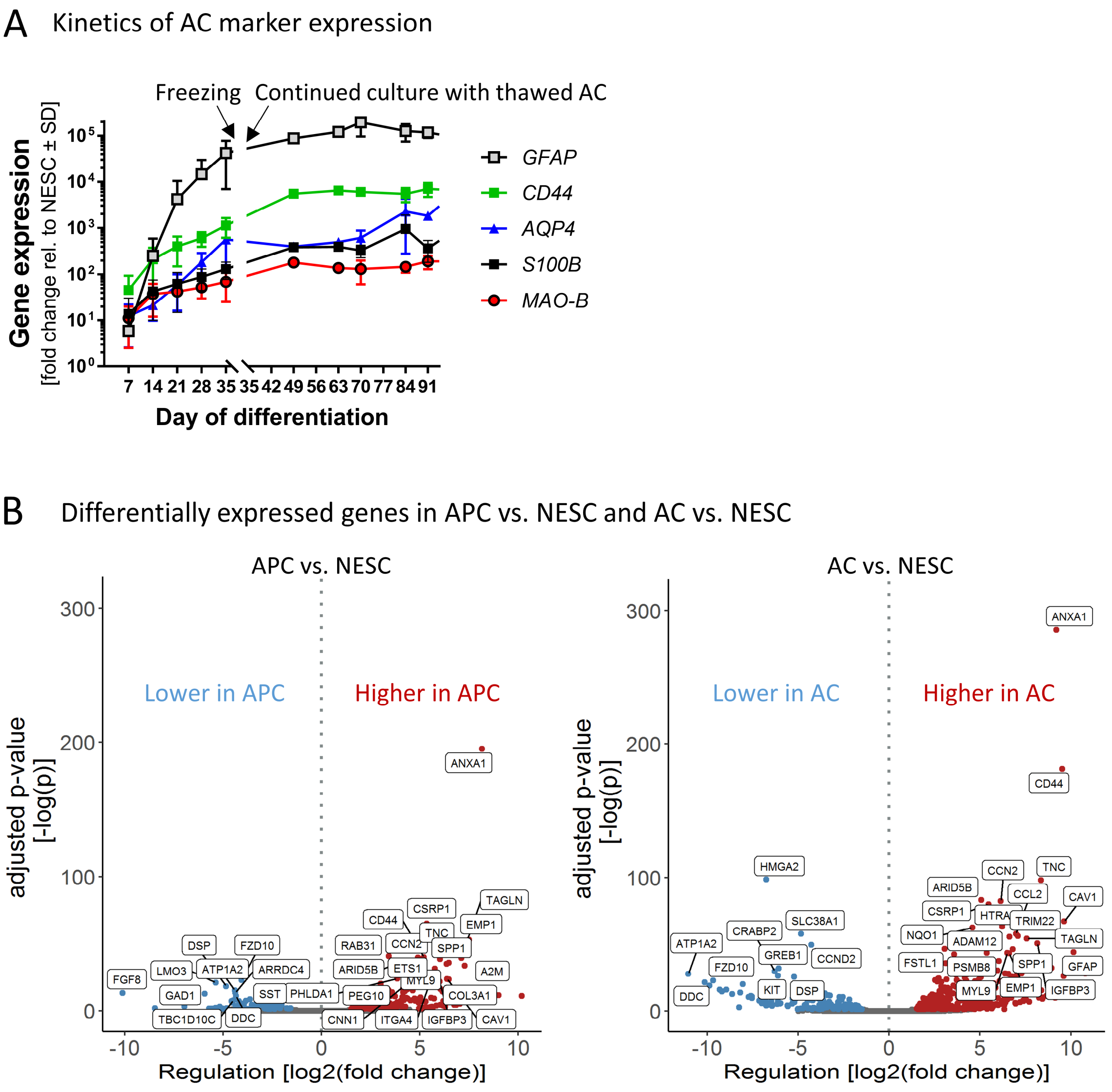

Figure S1. Gene expression of human astrocytes during differentiation. ACs were differentiated from neuroepithelial stem cells (NESCs) and expression changes of mRNA was followed for >100 days. The protocol involved three separate stages: (i) differentiation of NESCs to astrocyte progenitor cells (APCs) within 2 weeks (see Figure 1); (ii) differentiation of APCs to astrocytes (ACs) within 35 days, with freezing of cells thereafter; (iii) maturation of thawed ACs for further 63 days. (**A**) Five indicated AC markers (S100B, GFAP, CD44, AQP4 and MAO-B) were investigated by RT-qPCR gene expression analysis during the differentiation of APCs to fully matured ACs (98 days). Aliquots from parallel cultures were lysed and used for mRNA preparation and gene expression values are indicated relative to their expression in NESCs. Data are means ± SD of ≥ 3 independent differentiations. (**B**) The gene expression of APCs, ACs and NESC precursors was quantified for a panel of 3562 genes (four samples each) using targeted transcriptome analysis (TempOSeq method). Data was analyzed to identify differentially expressed genes (DEGs) of APCs (left plot) or ACs (right plot) vs. NESCs. Volcano plots show the statistical significance corrected for false discovery (adjusted p-value) in relation to the magnitude of regulation (log_2(fold change)) of every gene in the panel. DEGs are shown in blue if they were downregulated vs. NESCs ($p_{adj} < 0.05$, $log_2FC < -0.5$); and in red if they were upregulated vs. NESCs ($p_{adj} < 0.05$, $log_2FC > 0.5$). Nonregulated genes are shown in grey. The most significantly regulated genes are annotated (top 10 downregulated DEGs, top 20 upregulated DEGs).

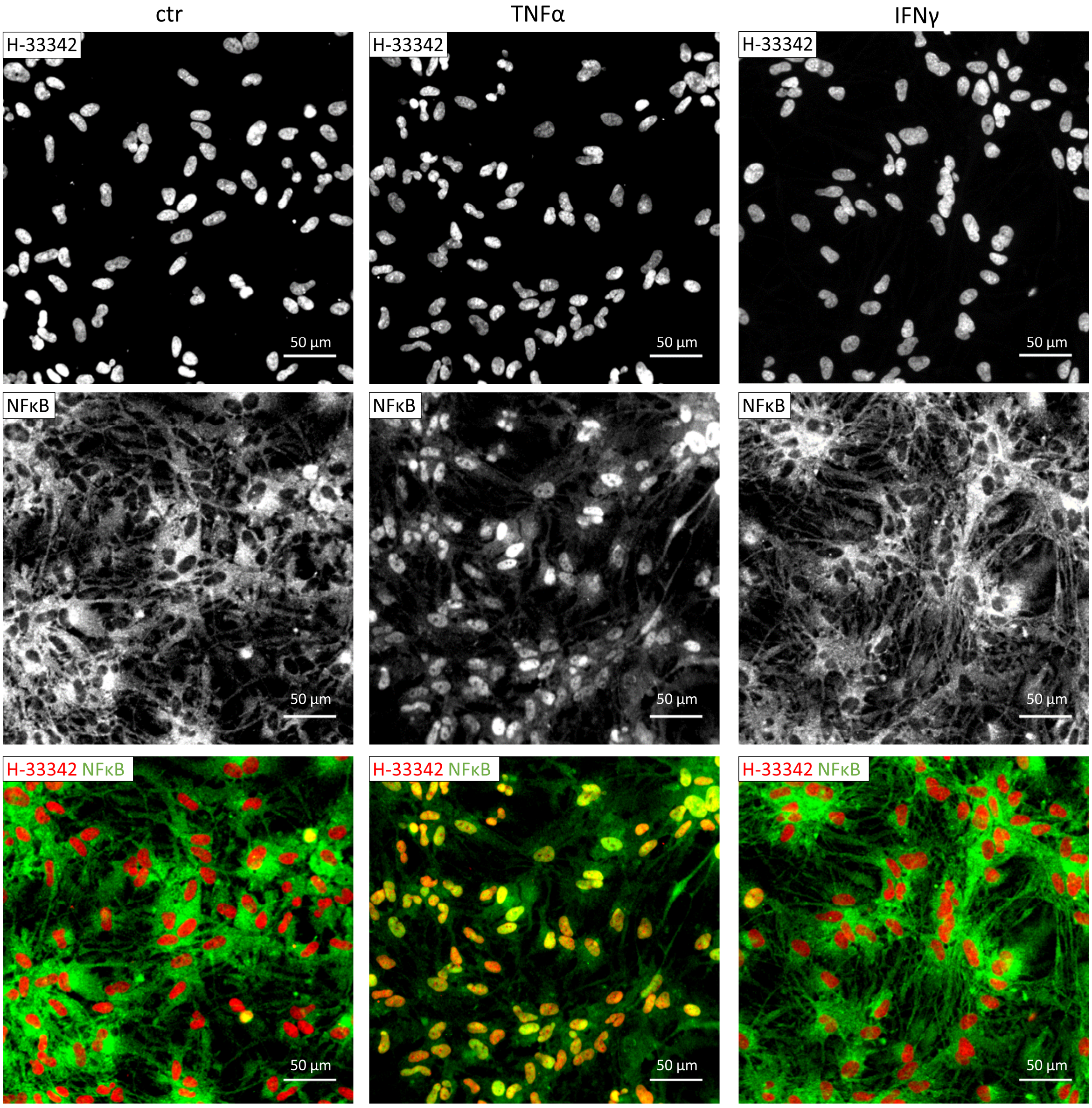

Figure S2. Translocation of NFκB in astrocytes after stimulation with cytokines. ACs were differentiated from APCs for more than 35 days. Three days after seeding (50,000 cells/cm²), cells were stimulated with cytokines: TNFα (10 ng/ml) or IFNγ (20 ng/ml). At 30 min after treatment, ACs were fixed and immunostained against the NFκB subunit p65. The nuclei were counterstained with Hoechst-33342. Exemplary images are shown which depict the same image fields as in Figure 2B, but give information on all fluorescent channels and their overlay.

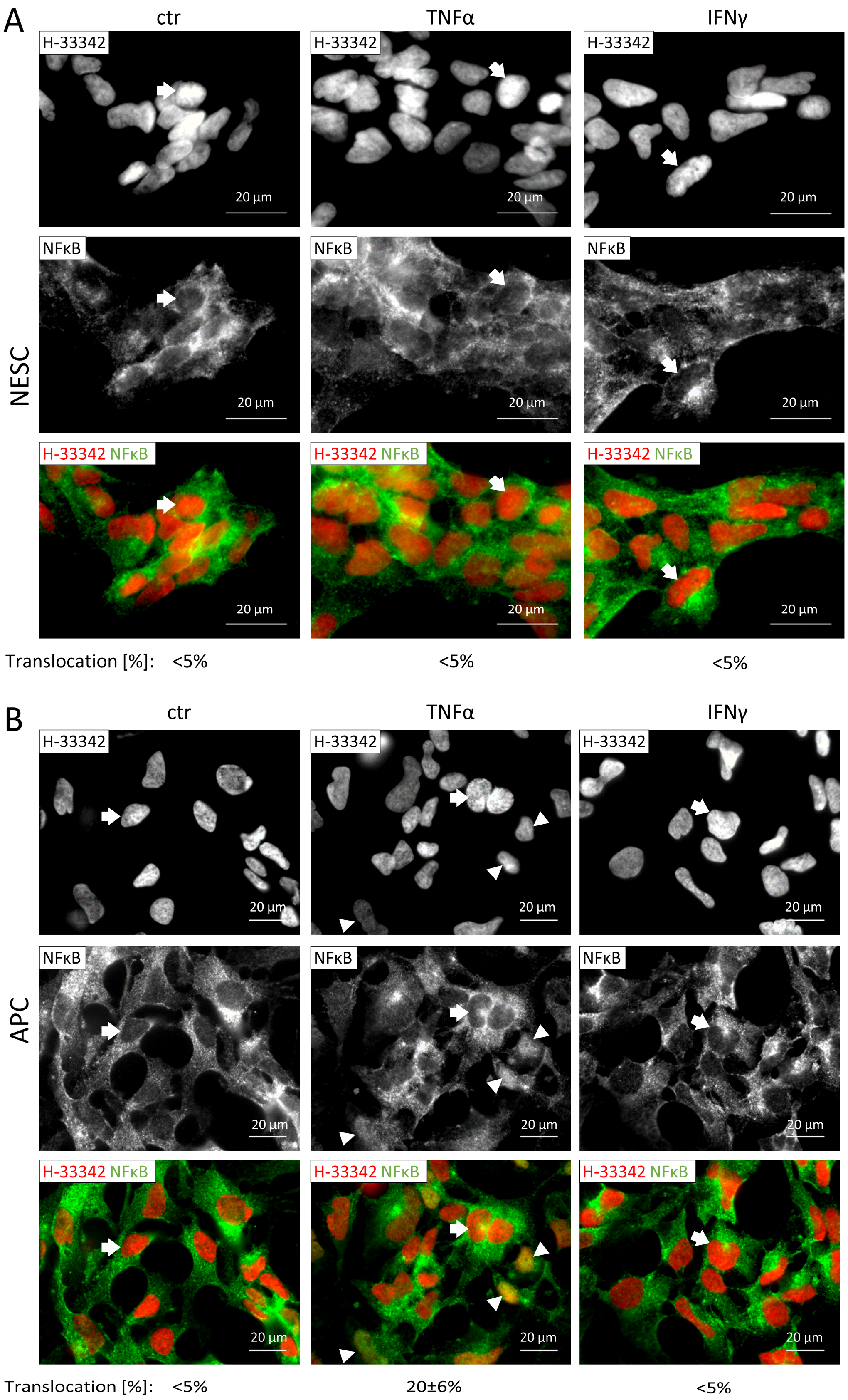

Figure S3. NFκB translocation in AC precursor populations after stimulation with cytokines. Neuroepithelial stem cells (NESCs) and astrocyte precursor cells (APCs) were differentiated from human iPSCs as described in Figure 1. At 24 h after seeding (50,000 cells/cm^2), the cells were stimulated with cytokines (TNFα (10 ng/ml) or IFNγ (20 ng/ml)). They were fixed 30 min later and immunostained against the NFκB subunit p65. The nuclei were counterstained with Hoechst H-33342. (**A**) The experiment was performed with NESCs and representative images are shown. Quantification of NFκB translocation was performed using an ArrayScan™ high-content

imaging device and a fully automated image recognition algorithm. Data are shown as the mean percentage of cells (n=3) with a nuclear NFκB p65 localization after cytokine stimulation. Arrows indicate exemplary nuclei in which NFκB p65 signals were absent (but cytosolic staining is visible). (**B**) The experiment was performed with APCs and representative images are shown. Quantification of NFκB translocation was performed using an ArrayScan™ high-content imaging device and a fully automated image recognition algorithm. Data are shown as the mean percentage of cells (n=3) with a nuclear NFκB p65 localization after cytokine stimulation. Arrows indicate nuclei in which NFκB p65 signals were absent (but cytosolic staining is visible). Arrowheads exemplify nuclei with NFκB p65 staining.

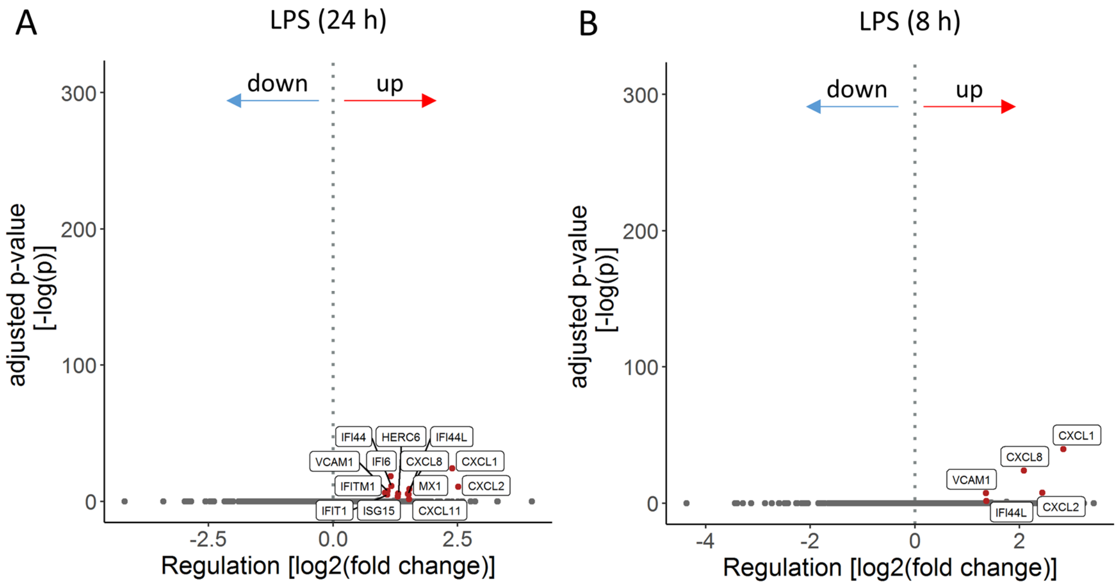

Figure S4. Transcriptional response of ACs to stimulation with LPS. ACs were seeded at a density of 50,000 cells/cm² into 96-well plates. On the next days, cells were stimulated with lipopolysaccharide from *Escherichia coli* O55:B5 (LPS; 100 ng/ml). After 8 h and 24 h, cells were lysed and gene expression profiling was performed for 3562 genes using RNASeq analysis (TempOSeq method). Data was analyzed to identify differentially expressed genes (DEGs) of ACs stimulated for (**A**) 8 h or (**B**) 24 h. The volcano plots show on the y-axis the statistical significance corrected for false discovery (adjusted p-value). The x-axis describes the magnitude of regulation fold change (FC) of every gene in the panel. Each DEG is represented by a grey dot in this plot. Some DEGs are shown in red. These were up-regulated vs. control (solvent) if the p-value was < 0.05 and FC > 1. All significantly regulated genes are annotated.

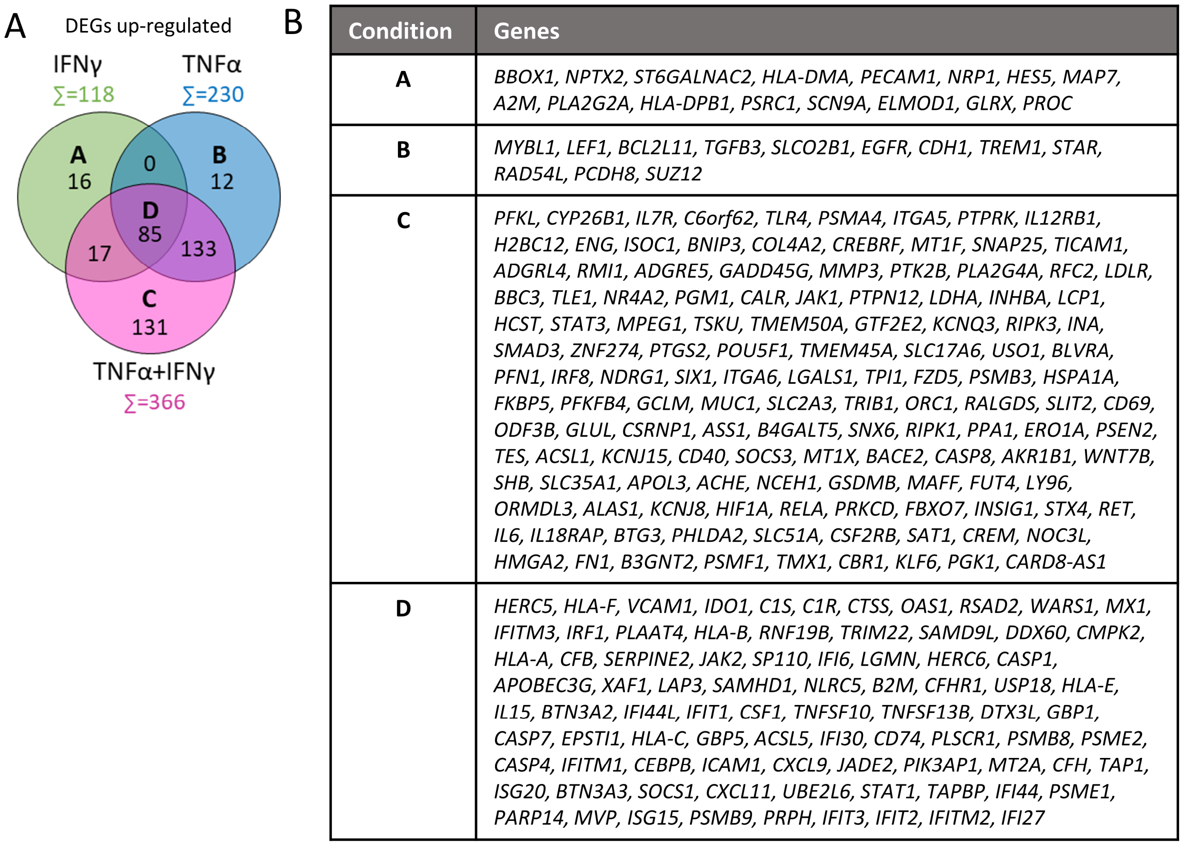

B

Condition	Genes
**A**	*BBOX1, NPTX2, ST6GALNAC2, HLA-DMA, PECAM1, NRP1, HES5, MAP7, A2M, PLA2G2A, HLA-DPB1, PSRC1, SCN9A, ELMOD1, GLRX, PROC*
**B**	*MYBL1, LEF1, BCL2L11, TGFB3, SLCO2B1, EGFR, CDH1, TREM1, STAR, RAD54L, PCDH8, SUZ12*
**C**	*PFKL, CYP26B1, IL7R, C6orf62, TLR4, PSMA4, ITGA5, PTPRK, IL12RB1, H2BC12, ENG, ISOC1, BNIP3, COL4A2, CREBRF, MT1F, SNAP25, TICAM1, ADGRL4, RMI1, ADGRE5, GADD45G, MMP3, PTK2B, PLA2G4A, RFC2, LDLR, BBC3, TLE1, NR4A2, PGM1, CALR, JAK1, PTPN12, LDHA, INHBA, LCP1, HCST, STAT3, MPEG1, TSKU, TMEM50A, GTF2E2, KCNQ3, RIPK3, INA, SMAD3, ZNF274, PTGS2, POU5F1, TMEM45A, SLC17A6, USO1, BLVRA, PFN1, IRF8, NDRG1, SIX1, ITGA6, LGALS1, TPI1, FZD5, PSMB3, HSPA1A, FKBP5, PFKFB4, GCLM, MUC1, SLC2A3, TRIB1, ORC1, RALGDS, SLIT2, CD69, ODF3B, GLUL, CSRNP1, ASS1, B4GALT5, SNX6, RIPK1, PPA1, ERO1A, PSEN2, TES, ACSL1, KCNJ15, CD40, SOCS3, MT1X, BACE2, CASP8, AKR1B1, WNT7B, SHB, SLC35A1, APOL3, ACHE, NCEH1, GSDMB, MAFF, FUT4, LY96, ORMDL3, ALAS1, KCNJ8, HIF1A, RELA, PRKCD, FBXO7, INSIG1, STX4, RET, IL6, IL18RAP, BTG3, PHLDA2, SLC51A, CSF2RB, SAT1, CREM, NOC3L, HMGA2, FN1, B3GNT2, PSMF1, TMX1, CBR1, KLF6, PGK1, CARD8-AS1*
**D**	*HERC5, HLA-F, VCAM1, IDO1, C1S, C1R, CTSS, OAS1, RSAD2, WARS1, MX1, IFITM3, IRF1, PLAAT4, HLA-B, RNF19B, TRIM22, SAMD9L, DDX60, CMPK2, HLA-A, CFB, SERPINE2, JAK2, SP110, IFI6, LGMN, HERC6, CASP1, APOBEC3G, XAF1, LAP3, SAMHD1, NLRC5, B2M, CFHR1, USP18, HLA-E, IL15, BTN3A2, IFI44L, IFIT1, CSF1, TNFSF10, TNFSF13B, DTX3L, GBP1, CASP7, EPSTI1, HLA-C, GBP5, ACSL5, IFI30, CD74, PLSCR1, PSMB8, PSME2, CASP4, IFITM1, CEBPB, ICAM1, CXCL9, JADE2, PIK3AP1, MT2A, CFH, TAP1, ISG20, BTN3A3, SOCS1, CXCL11, UBE2L6, STAT1, TAPBP, IFI44, PSME1, PARP14, MVP, ISG15, PSMB9, PRPH, IFIT3, IFIT2, IFITM2, IFI27*

Figure S5. Genes uniquely expressed in ACs stimulated with cytokines for 24 h. (A) Venn diagram depicting the number of genes up-regulated in ACs ≥ 2-fold and with $p_{adj} < 0.05$. Numbers in overlapping areas represent the genes commonly regulated. **(B)** Genes uniquely expressed in ACs stimulated with TNFα only (A), IFNγ only (B), co-treatment with TNFα and IFNγ (C) or genes overlapping between all conditions (D).

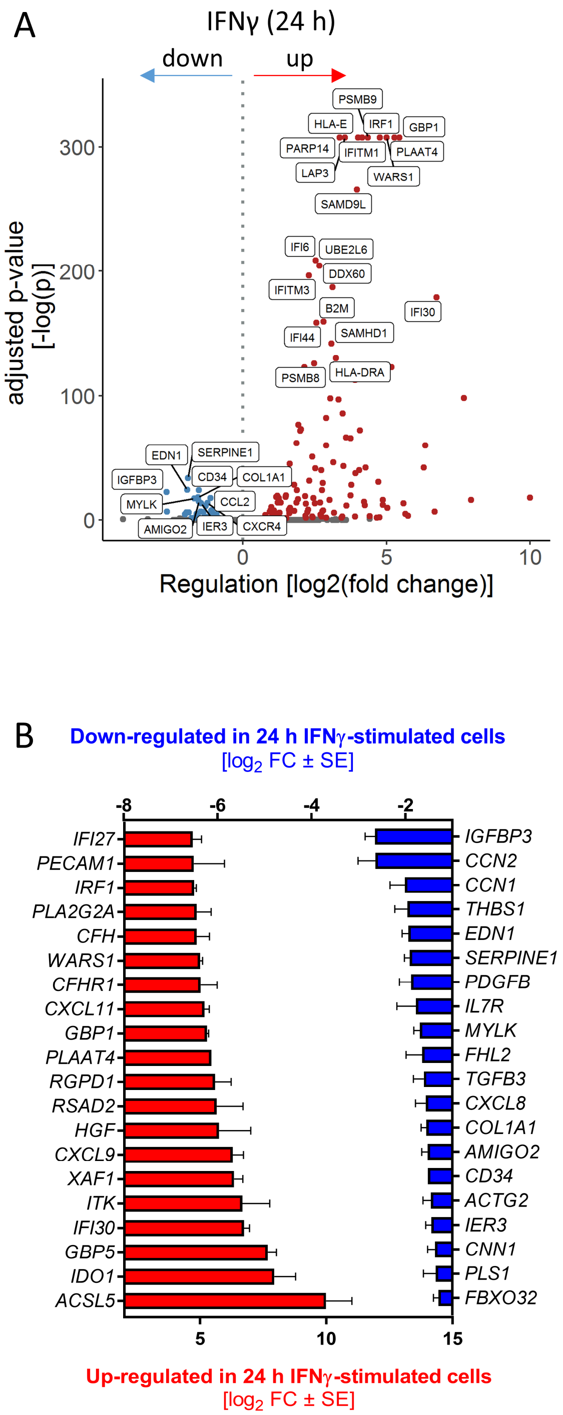

Figure S6. Transcriptional response of ACs to IFNγ stimulation for 24 h. ACs were seeded at a density of 50,000 cells/cm^2 into 96-well plates. Three days after seeding, cells were stimulated with IFNγ (20 ng/ml). After 24 h, cells were lysed and gene expression profiling was performed for 3562 genes using RNASeq analysis (TempOSeq method). Data was analyzed to identify differentially expressed genes (DEGs) of stimulated ACs relative to control ACs. (**A**) Volcano plots show the statistical significance corrected for false discovery (adjusted p-value (p_{adj})) in relation to the magnitude of regulation (log_2(fold change)) of every gene in the panel. DEGs are shown in blue, if they were downregulated vs. control ACs (p_{adj} <0.05, log_2FC < -0.5); and in red if they were upregulated vs. control ACs (p_{adj} <0.05, log_2FC > 0.5). Non-regulated genes are shown in grey. The top 20 up/down-regulated DEGs are annotated. (**B**) Bar graphs, showing the top 20 up- and down-regulated DEGs of ACs stimulated with IFNγ for 24 h. The error bars represent the standard deviation. Data are means ± SE, n=4.

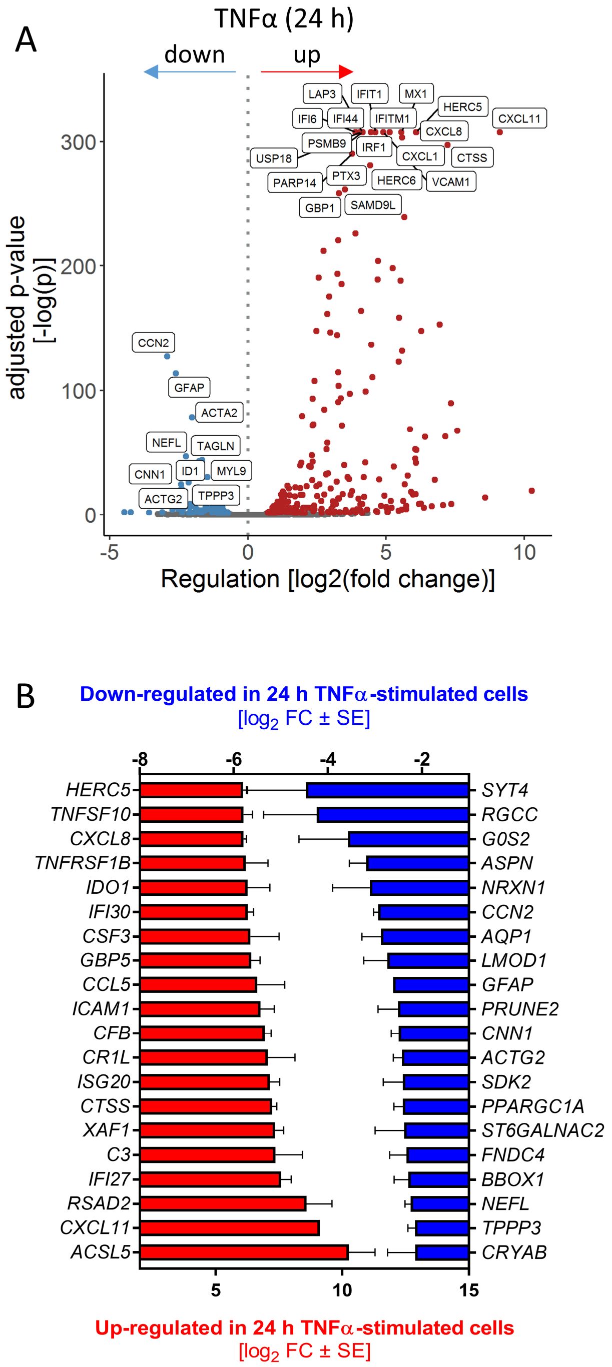

Figure S7. Transcriptional response of ACs to TNFα stimulation for 24 h. ACs were seeded at a density of 50,000 cells/cm² into 96-well plates. Three days after seeding, cells were stimulated with TNFα (10 ng/ml). After 24 h, cells were lysed and gene expression profiling was performed for 3562 genes using RNASeq analysis (TempOSeq method). Data was analyzed to identify differentially expressed genes (DEGs) of stimulated ACs relative to control ACs. (**A**) Volcano plots show the statistical significance corrected for false discovery (adjusted p-value (p_{adj})) in relation to the magnitude of regulation (log_2(fold change)) of every gene in the panel. DEGs are shown in blue, if they were downregulated vs. control AC (p_{adj} <0.05, log_2FC < -0.5); and in red if they were upregulated vs. control ACs (p_{adj} <0.05, log_2FC > 0.5). Non-regulated genes are shown in grey. The top 20 up/down-regulated DEGs are annotated. (**B**) Bar graphs, showing the top 20 up- and down-regulated DEGs of ACs stimulated with TNFα for 24 h. The error bars represent the standard deviation. Data are means ± SE, n=4.

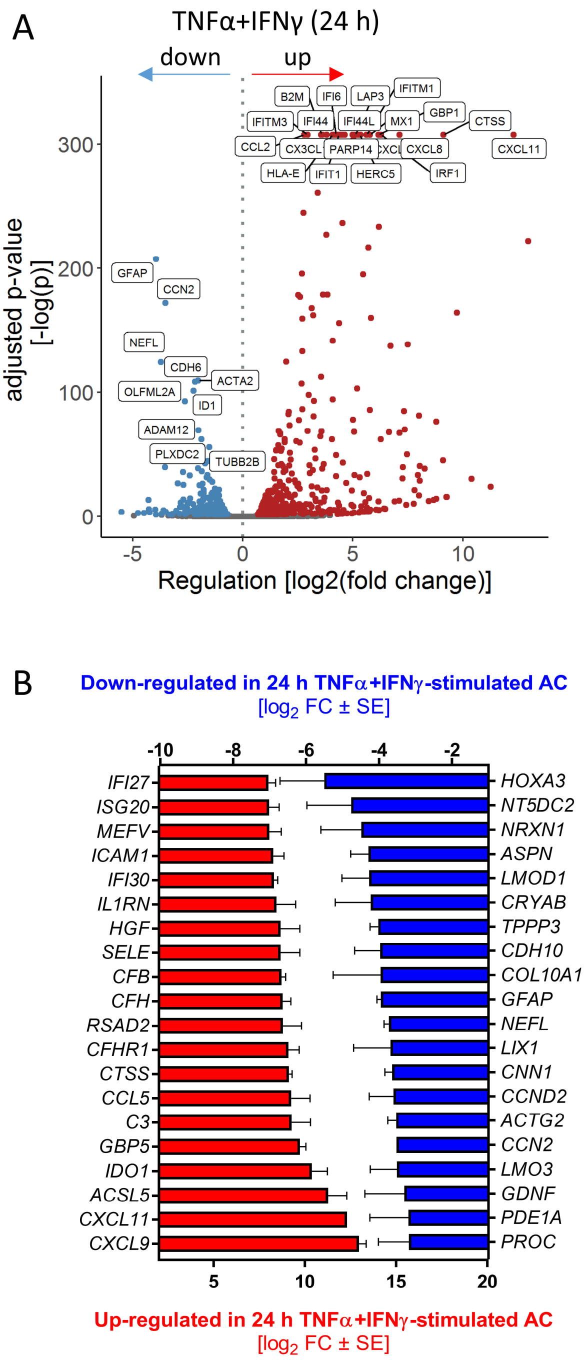

Figure S8. Transcriptional response of ACs to combined treatment with TNF*α* *plus* IFNγ for 24 h. ACs were seeded at a density of 50,000 cells/cm² into 96-well plates. Three days after seeding, cells were stimulated with TNF*α* (10 ng/ml) and IFNγ (20 ng/ml). After 24 h, cells were lysed and gene expression profiling was performed for 3562 genes using RNASeq analysis (TempOSeq method). Data was analyzed to identify differentially expressed genes (DEGs) of stimulated ACs relative to control AC. (**A**) Volcano plots show the statistical significance corrected for false discovery (adjusted p-value (p_{adj})) in relation to the magnitude of regulation (log_2(fold change)) of every gene in the panel. DEGs are shown in blue, if they were downregulated vs. control ACs (p_{adj} <0.05, log_2FC < -0.5); and in red if they were upregulated vs. control AC (p_{adj} <0.05, log2FC > 0.5). Non-regulated genes are shown in grey. The top 20 up/down-regulated DEGs are annotated. (**B**) Bar graphs, showing the top 20 up- and down-regulated DEGs of ACs stimulated with TNF*α* *plus* IFNγ for 24 h. The error bars represent the standard deviation. Data are means ± SE, n=4.

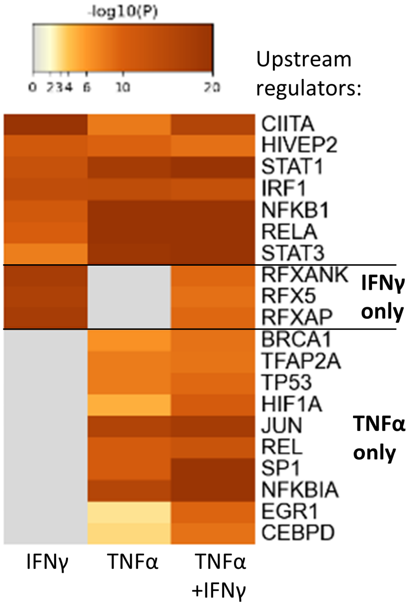

Figure S9. Overrepresentation of transcription factor binding sites amongst ACs regulated by cytokines. Transcription factors (TF) whose binding sites were over-represented on promoters of up-regulated DEGs (p_{adj} <0.05, FC ≥ 2) in ACs stimulated for 24 h with TNFα (10 ng/ml), IFNγ (20 ng/ml) or the combination of TNFα (10 ng/ml) *plus* IFNγ (20 ng/ml) were identified using Metascape [1] and labelled here as “upstream regulators”. Shown are the top 20 key regulators based on the TRRUST database (v.2) according to p-value. They are clustered by p-value at each treatment condition. Cytokines with unique activation of TF are implicated to the right. The p-value metrics are indicated by a colour code (darker brown intensity = lower p-value = higher over-representation). Grey fields code for non-over-represented TF at the respective stimulation condition.

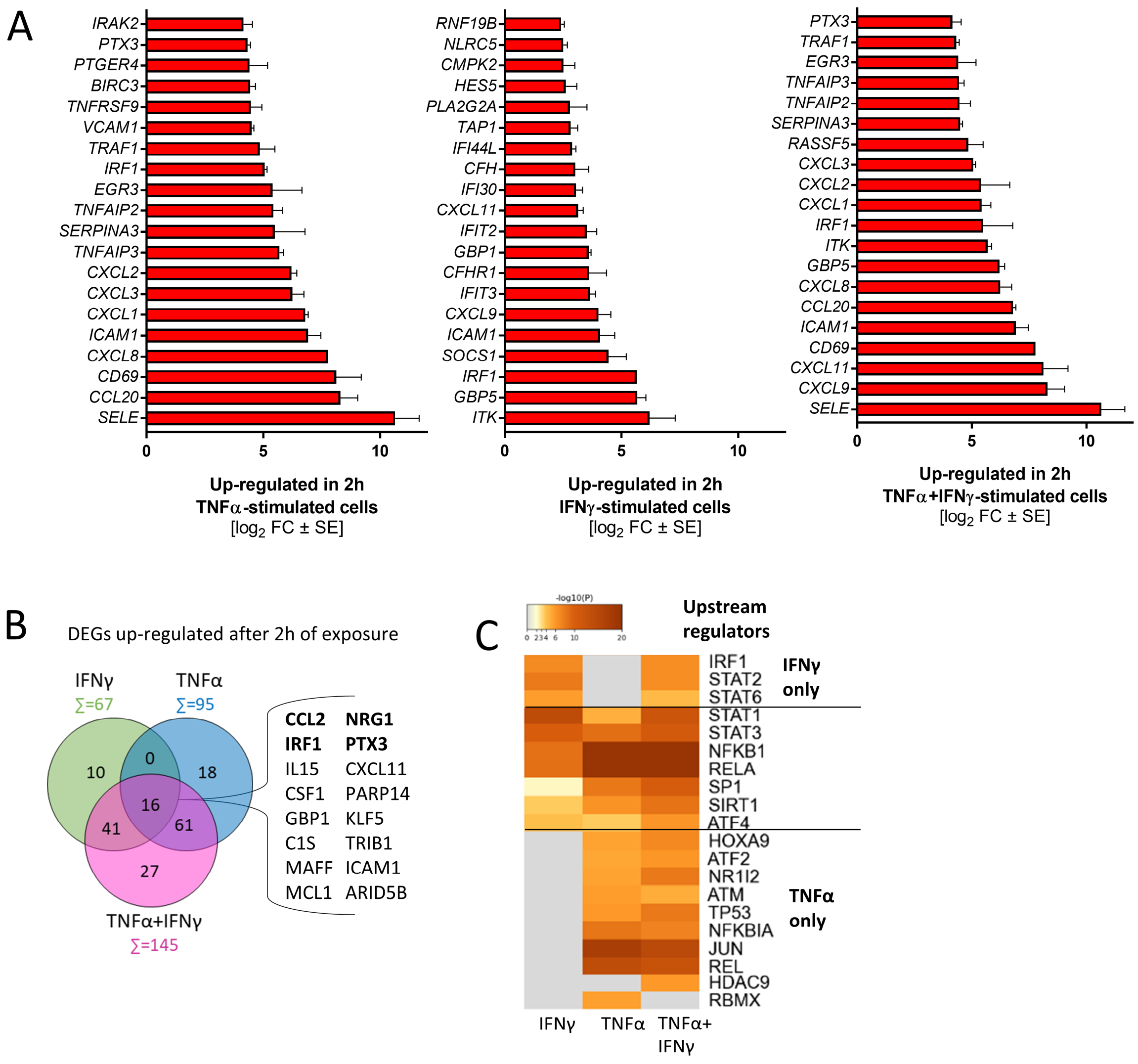

Figure S10. Response of ACs to cytokines upon stimulation for 2 h. ACs were seeded at a density of 50,000 cells/cm² into 96-well plates. Four days after seeding, cells were stimulated with TNFα (10 ng/ml), IFNγ (20 ng/ml) or a combination of TNFα (10 ng/ml) *plus* IFNγ (20 ng/ml). After 2 h, cells were lysed and gene expression profiling was performed for 3562 genes. (**A**) Bar graphs depicting the top 20 up-regulated genes in TNFα stimulated ACs (left blot), IFNγ stimulated ACs (middle) and TNFα *plus* IFNγ co-stimulated ACs (right). The error bars represent the standard deviation. Data are means ± SE, n=4. (**B**) Venn diagram depicting the number of genes up-regulated in ACs ≥ 2-fold and with $p_{adj} < 0.05$. Numbers in overlapping areas represent the genes commonly regulated, indicated are the genes which overlap between all treatment conditions. (C) Transcription factors (TF) whose binding sites were over-represented on promoters of up-regulated DEGs (p_{adj} <0.05, FC ≥ 2) in ACs stimulated for 2 h with TNFα (10 ng/ml), IFNγ (20 ng/ml) or the combination of TNFα (10 ng/ml) and IFNγ (20 ng/ml) were identified using Metascape [1] and labelled here as "upstream regulators". Shown are the top 20 key regulators based on the TRRUST database (v.2) according to p-value. They are clustered by p-value at each treatment condition. Cytokines with unique activation of TF are implicated to the right. The p-value metrics are indicated by a color code (darker brown intensity = lower p-value = higher over-representation). Grey fields code for non-over-represented TF at the respective stimulation condition.

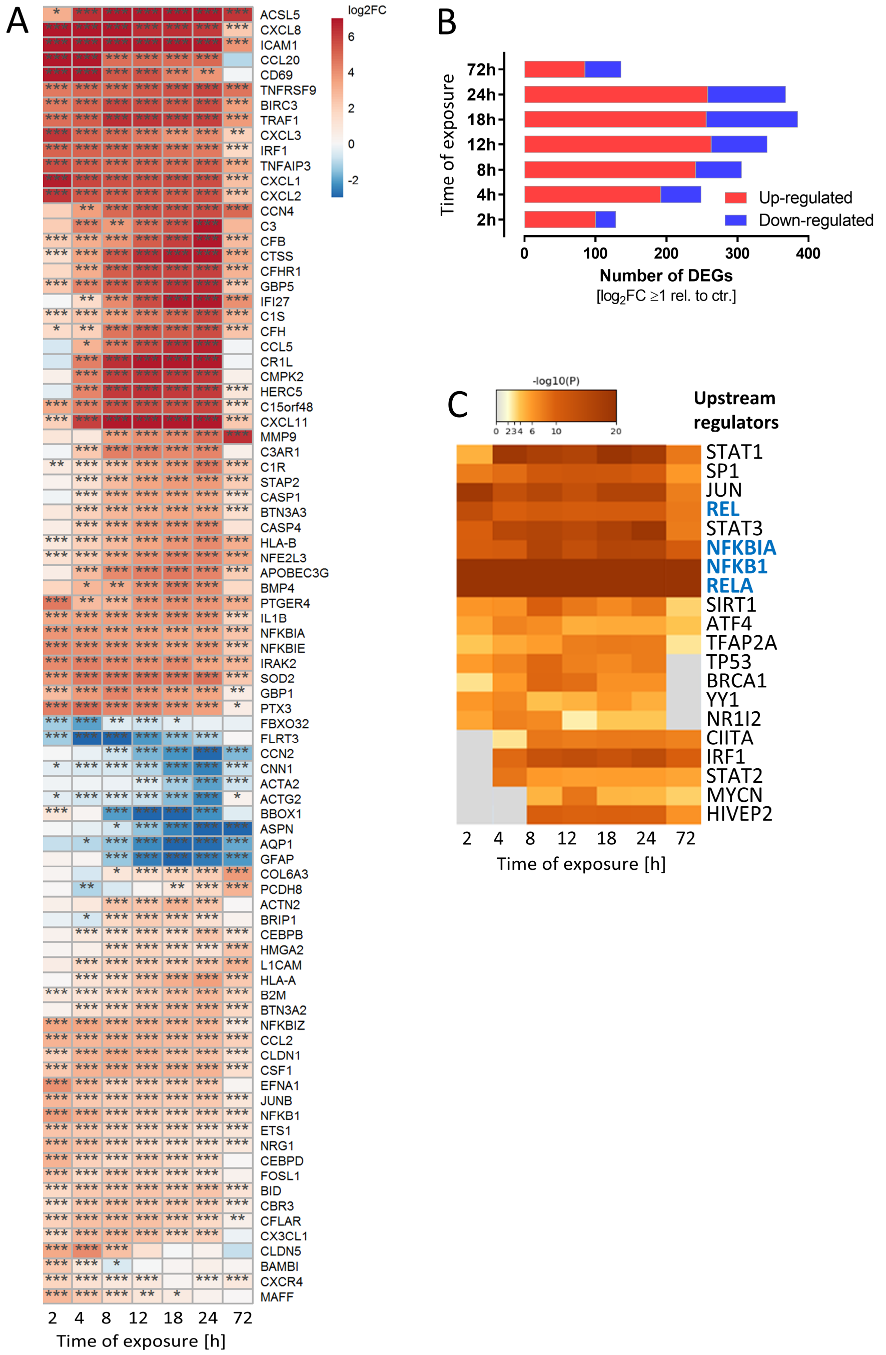

Figure S11. Time-dependent transcriptome response of ACs to TNFα stimulation. ACs were seeded at a density of 50,000 cells/cm² into 96-well plates. On the following days, cells were stimulated with TNFα (10 ng/ml). After indicated time periods, cells were lysed and gene expression profiling was performed for 3562 genes. (**A**) Heat map showing the top regulated genes across all time points after treatment with TNFα. The top 30 most regulated genes were identified for each time point and merged to a common gene list. The $\log_2$ fold change ($\log_2$FC) against untreated cells is shown (red: up-regulation; blue: down-regulation). Row-wise clustering was performed to group genes with similar regulation patterns. Asterisks indicate the FDR-corrected statistical significance of the expression *vs.* untreated control cells. *$p_{adj} < 0.05$, **$p_{adj} < 0.01$, ***$p_{adj} < 0.001$. (**B**) The total number of differentially expressed genes (DEGs) are depicted. Genes were considered as differentially expressed with p-value ≤ 0.05 (after false discovery rate adjustment) and an absolute $\log_2$fold change of ≥ 0.5. (**C**) Transcription factors (TF) whose binding sites were over-represented on promoters of up-regulated DEGs (p_{adj} <0.05, FC ≥ 2) in ACs stimulated for the indicated time periods with TNFα (10 ng/ml) were identified using Metascape [1], and labelled here as "upstream regulators". Shown are the top 20 key regulators based on the TRRUST database (v.2) according to p-value. They are clustered by p-value at each treatment condition. Cytokines with unique activation of TF are implicated to the right. The p-value metrics are indicated by a color code (darker brown intensity = lower p-value = higher over-representation). Grey fields code for non-over-represented TF at the respective stimulation condition.

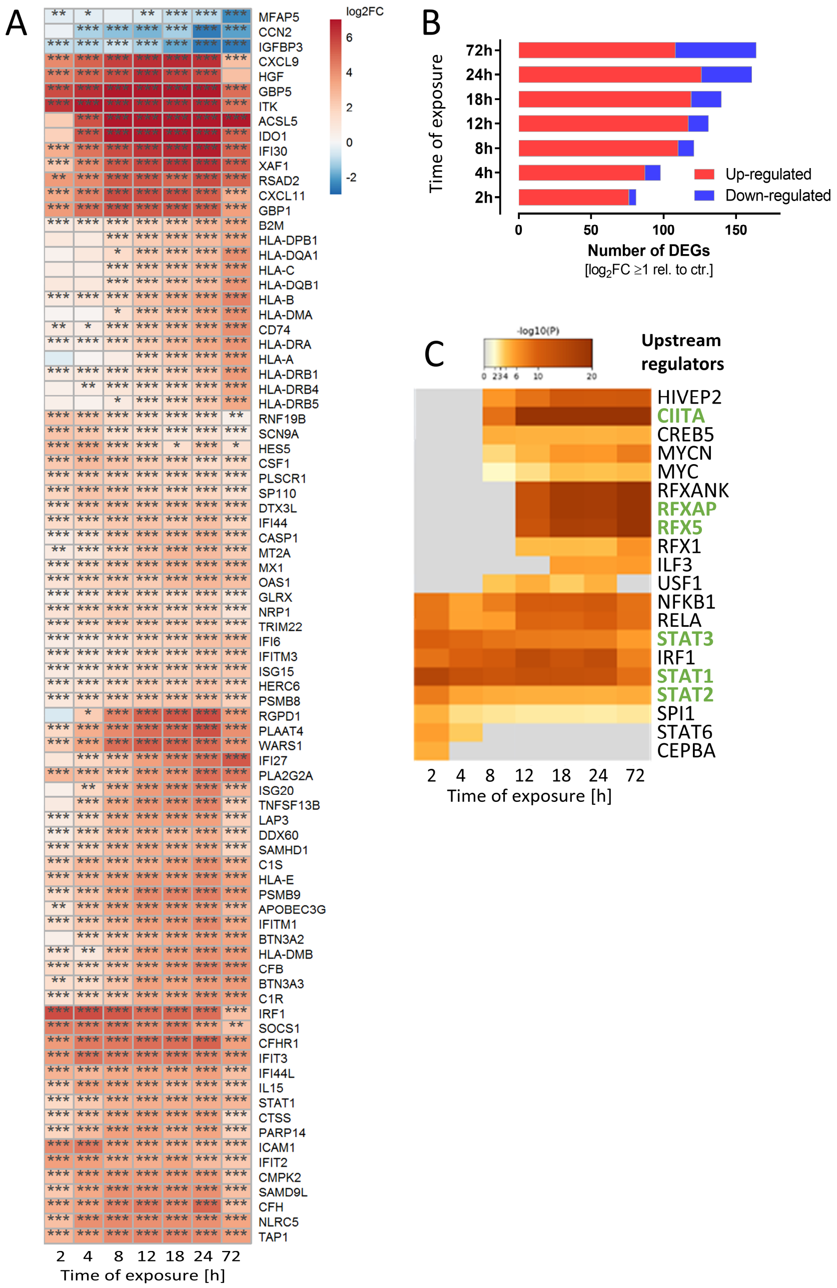

Figure S12. Time-dependent transcriptome response of ACs to IFNγ stimulation. ACs were seeded at a density of 50,000 cells/cm² into 96-well plates. On the following days, cells were stimulated with IFNγ (20 ng/ml). After indicated time periods, cells were lysed and gene expression profiling was performed for 3562 genes. (**A**) Heat map showing the top regulated genes across all time points after treatment with IFNγ. The top 30 most regulated genes were identified for each time point and merged to a common gene list. The $\log_2$ fold change ($\log_2$FC) against untreated cells is shown (red: up-regulation; blue: down-regulation). Row-wise clustering was performed to group genes with similar regulation patterns. Asterisks indicate the FDR-corrected statistical significance of the expression vs. untreated control cells. *$p_{adj} < 0.05$, **$p_{adj} < 0.01$, ***$p_{adj} < 0.001$. (**B**) The total number of differentially expressed genes (DEGs) are depicted. Genes were considered as differentially expressed with p-value ≤ 0.05 (after false discovery rate adjustment) and an absolute $\log_2$fold change of ≥ 0.5. (**C**) Transcription factors (TF) whose binding sites were over-represented on promoters of up-regulated DEGs (p_{adj} <0.05, FC ≥ 2) in ACs stimulated for the indicated time periods with IFNγ (20 ng/ml) were identified using Metascape [1], and labelled here as "upstream regulators". Shown are the top 20 key regulators based on the TRRUST database (v.2) according to p-value. They are clustered by p-value at each treatment condition. Cytokines with unique activation of TF are implicated to the right. The p-value metrics are indicated by a colour code (darker brown intensity = lower p-value = higher over-representation). Grey fields code for non-over-represented TF at the respective stimulation condition.

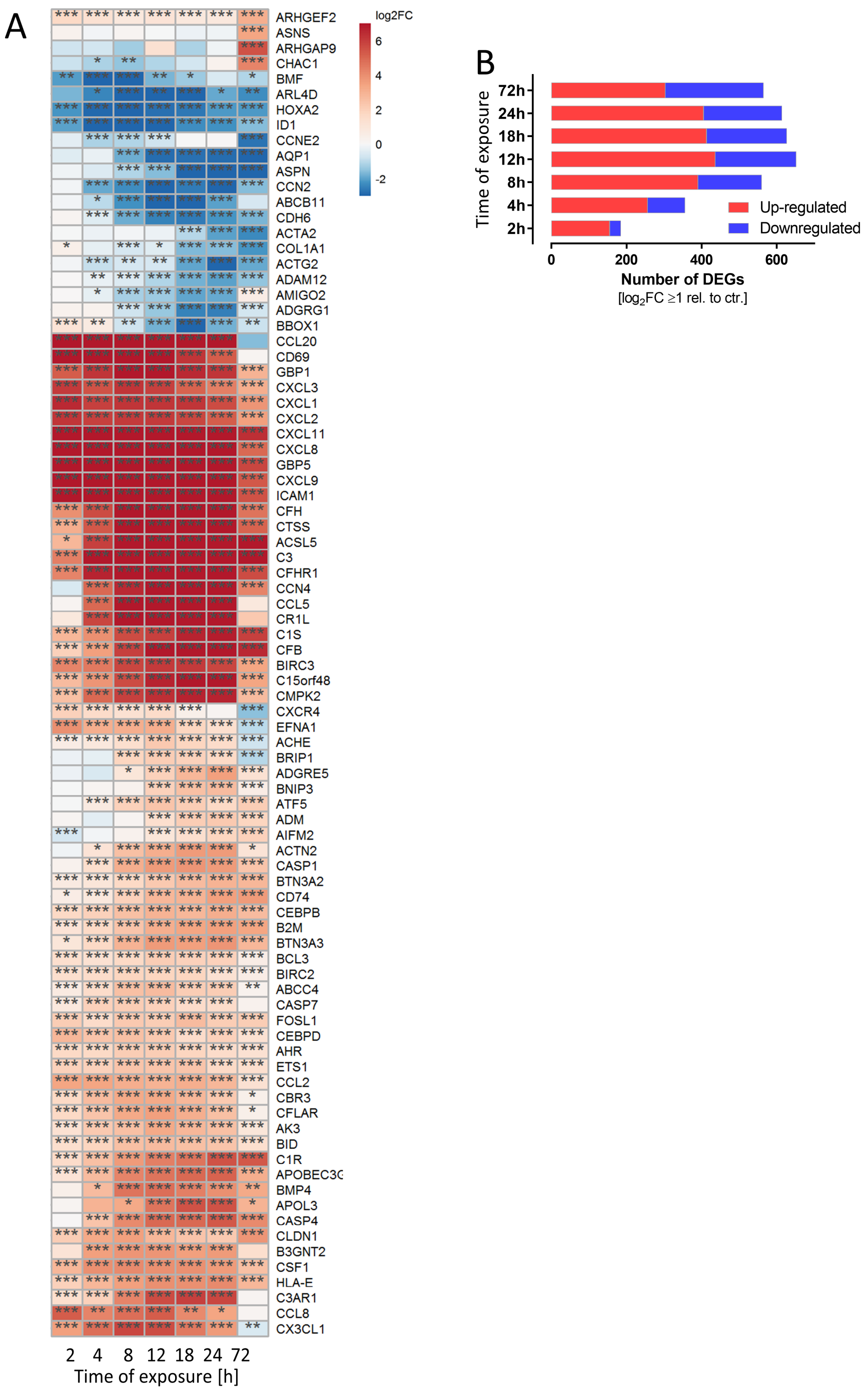

Figure S13. Time-dependent transcriptome response of ACs to combined TNFα *plus* IFNγ stimulation. ACs were seeded at a density of 50,000 cells/cm^2 into 96-well plates. On the following days, cells were stimulated with TNFα (10 ng/ml) and IFNγ (20 ng/ml). After indicated time periods, cells were lysed and gene expression profiling was performed for 3562 genes. (**A**) Heat map showing the top regulated genes across all time points after combined treatment with TNFα and IFNγ. The top 30 most regulated genes were identified for each time point and merged to a common gene list. The $\log_2$ fold change ($\log_2$FC) against untreated cells is shown (red: up-regulation; blue: down-regulation). Row-wise clustering was performed to group genes with similar regulation patterns. Asterisks indicate the FDR-corrected statistical significance of the expression *vs*. untreated control cells. *$p_{adj} < 0.05$, **$p_{adj} < 0.01$, ***$p_{adj} < 0.001$. (**B**) The total number of differentially expressed genes (DEGs) are depicted. Genes were considered as differentially expressed with p-value ≤ 0.05 (after false discovery rate adjustment) and an absolute $\log_2$fold change of ≥ 0.5.

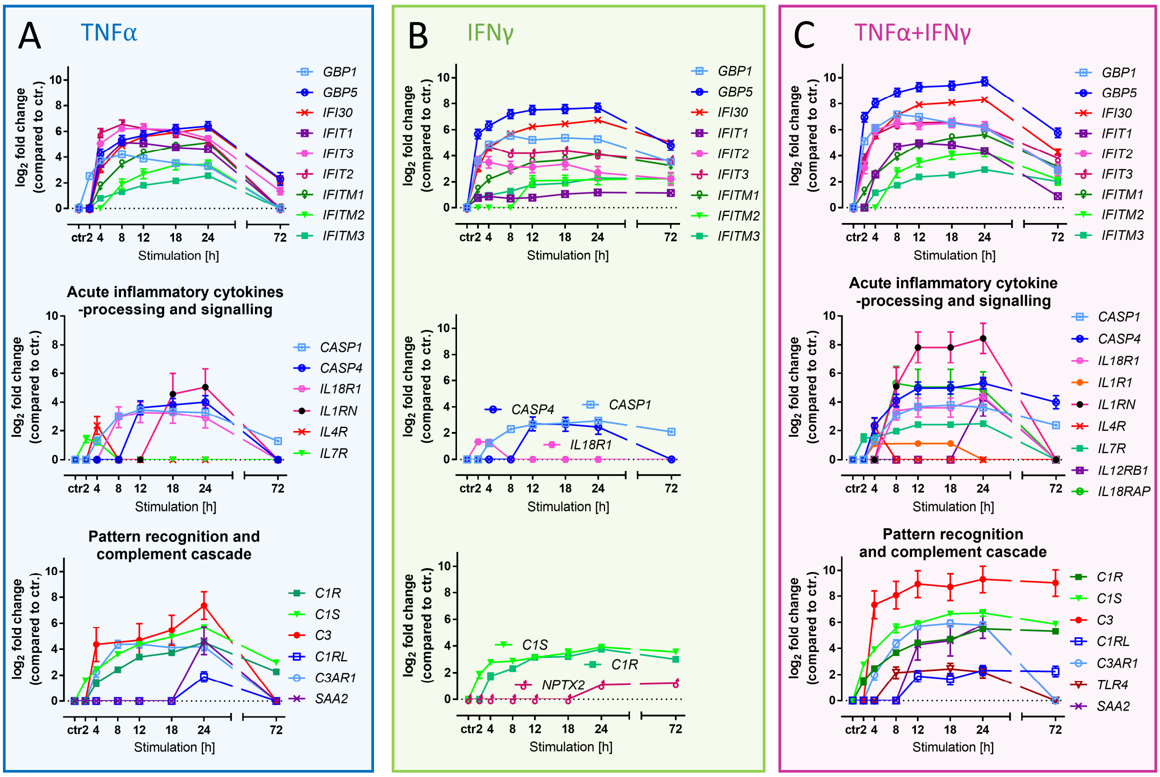

Figure S14. Differential regulation of additional genes coding for inflammation markers. ACs were seeded at a density of 50,000 cells/cm² into 96-well plates. On the following days, cells were stimulated with (**A**) TNFα (10 ng/ml), (**B**) IFNγ (20 ng/ml) or the combination of (**C**) TNFα (10 ng/ml) *plus* IFNγ (20 ng/ml). After indicated time periods, cells were lysed and gene expression profiling was performed for 3562 genes. Depicted are levels of mRNA for exemplary proteins involved in diverse inflammation processes. All data are means ± SE from four samples (two samples each from 2 independent differentiations). Ctr.: unstimulated control ACs.

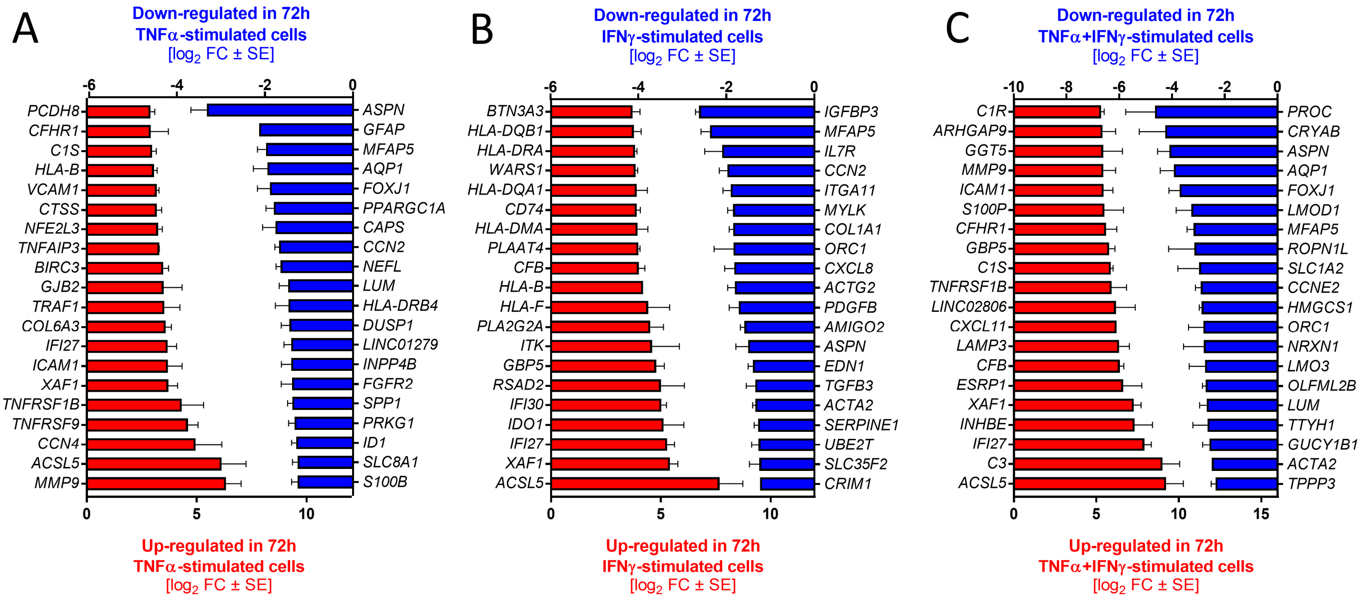

Figure S15. Response of ACs to cytokines upon stimulation for 72 h. ACs were seeded at a density of 50,000 cells/cm² into 96-well plates. On the next day, cells were stimulated with TNFα (10 ng/ml), IFNγ (20 ng/ml) or the combination of TNFα (10 ng/ml) *plus* IFNγ (20 ng/ml). After 72 h, cells were lysed and gene expression profiling was performed for 3562 genes. Bar graphs depicting the top 20 up-regulated genes in (**A**) TNFα stimulated AC, (**B**) IFNγ stimulated AC and (**C**) TNFα *plus* IFNγ co-stimulated AC. Data are means ± SE, n=4.

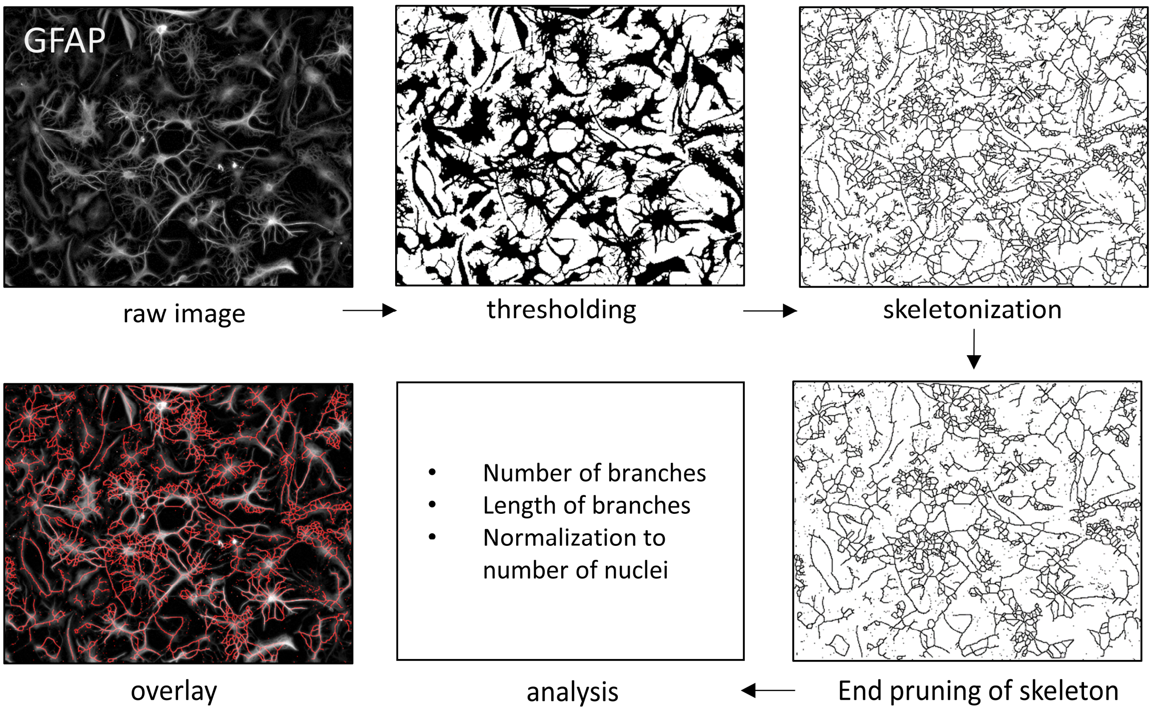

Figure S16. Schematic workflow for the quantification of morphological changes in AC stimulated with cytokines. For quantification of astrocyte morphology after inflammatory stimulation, images of immunostained astrocytes (GFAP, H-33342) were analysed with FIJI (version 1.53f51). Images were background corrected and enhanced with a decreased gamma value. The minimum pixel value was increased until cell-free areas appeared as black ("corrected image"). To obtained binary images, images were then auto-thresholded ("thresholding"). Binary images of the GFAP channel were iteratively reduced to lines with FIJI's built-in "skeletonize" function ("skeletonization"). Skeletons were pruned to avoid overestimation of branching of cells without distinct processes ("End pruning of skelton"). The skeletonized images were analysed with the built-in "analyze skeleton" function. To obtain the number of cells per field, binary images of the H-33342-channel were analysed. Particles >10 µm² were counted as nuclei. The total length of branches and the number of branches in each field was normalized to the number of nuclei of the respective field. The bottom left panel shows the skeletonized images over the corrected image.

Table S1. Additional information on differentiation

	Growth Morphology	Seeding density for culture	Seeding density for differentiation	Proliferation rate	Use period
NESC	Cells gather in colonies. Some spontaneously differentiated neurons can be detected especially on the borders of the colonies	20-40 *k* NESC/cm^2 for culture; passaging after 3-4 days	50 *k* NESC/ cm^2 for differentiation to APC	High:<br>6-8-fold within 4 days	Frozen in P3 (day 25 of differentiation from iPSC) [2, 3]<br><br>P3-P10 can be used for differentiation to APC*
APC	Cells are evenly distributed across the well, do not gather in colonies compared to NESC, can get confluent	20 *k* APC/cm^2 for passaging after 4 days;<br>40 *k* APC/cm^2 for passaging after 3 days	20-50 *k* APC/cm^2 for differentiation to AC	High:<br>6-fold within 4 days,<br>3-fold within 3 days	APC are suitable for AC differentiation already from day 11 – 18*. Can be frozen on day 7 and thawed for AC differentiation
AC	Cells are evenly distributed across the well; form “networks”	100 *k* AC/cm^2 until day 35 of differentiation.<br><br>After d35: 60 *k* AC/cm^2 or less to accelerate proliferation rate and to increase the amount of cells for further experiments.<br><br>Passaging is performed once a week		Low:<br>less than 2-fold per week.<br>Can be further reduced by serum-withdrawal	Have been cultured up to 100 days

k = Kilo = 10^3

P = Passage

*might need adaptations for different iPSC lines

Critical Steps for AC differentiation/ culture	• AC need some cell-cell contact, especially in the first 5 weeks of differentiation from APC<br>• Proliferation rate gets lower as a sign of successful differentiation:<br>• 2.5-fold (doubling in one week) in the first 2 weeks of differentiation, 1.5 to 2-fold doubling from day 14 to d 21, 1.2-fold on d 35<br>• Stable or rising proliferation rates indicate wrong differentiation!<br>• AC (d 35) stain homogeneously positive for CD44, VIM, S100B; majority (>85%) stain positive for GFAP.
Non-critical steps for AC differentiation	• APC can be frozen on day 7 or be amplified for up to 5 passages before AC differentiation<br>• Frozen/thawed APC can be used for AC differentiation<br>• start of AC differentiation with 20 - 50 *k* APC/cm^2 possible, resulting in similar marker expression on day 35 of AC differentiation<br>• Small cell aggregates can be detected while splitting. They can be dissolved by longer incubation time with Accutase or by using a cell strainer to achieve single cells only; it might be necessary to have defined cell densities for experiments<br>• The content of serum in the medium can be reduced to 0% for experiments (after 35 days of differentiation from APC), co-cultivation of AC with neurons in neuronal medium (0% serum) is also possible.

Table S2. Comparison of markers with TIC-induced astrocytes.

TIC-induced reactive AC marker (Labib et al. [4])	Treatment condition		
	**TNFα**	**IFNγ**	**TNFα + IFNγ**
IFI6	+	+	+
MX1	+	+	+
ICAM1	+	+	+
GBP1	+	+	+
IFIT1	+	+	+
IFIT3	+	+	+
HLA-F	+	+	+
CTSS	+	+	+
IFIT2	+	+	+
CXCL11	+	+	+
ISG15	+	+	+
C1R	+	+	+
WARS1	+	+	+
STAT1	+	+	+
OAS1	+	+	+
C15orf48	+	-	+
SOD2	+	-	+
CCL2	+	-	+
TNFAIP2	+	-	+
TRAF1	+	-	+
CXCL1	+	-	+
CCL5	+	-	+
CYP1B1	+	-	+
PHOSPHO1	-	-	-

Table S3. Overview of genes uniquely differentially expressed (DEGs) in ACs stimulated with cytokines for 72 h

Condition	Number of DEGs	Names of DEGs
IFNy only	25	*BBOX1, NPTX2, CEBPD, ST6GALNAC2, SULF1, HLA-DMB, MAP7, A2M, PLA2G2A, TPPP3, RGPD1, IFIT1, ITK, HLA-DPB1, CLU, HLA-DRB3, SOCS3, SEMA4B, CA2, PSRC1, PIFO, SEMA6A, PRPH, TMED10, GFAP*
TNFα only	9	*LEF1, BMF, TGFB3, ROBO1, JUNB, PODXL, NRIP1, FN1, ITGA4*
TNFα+IFNγ combined treatment	162	*MT1G, ESRP1, PSMA4, CSTA, ITGA5, RGS2, SUPV3L1, PTPRK, H2BC12, IARS1, AEN, KCNG1, PLAU, MTHFD2, CXCL3, SQOR, FTH1, TP53, GDF15, GFPT1, HMOX1, C1RL, GRB10, PPP1R15A, P4HA2, S100P, C15orf48, ADGRL4, TRIB3, STXBP2, FUT1, IGF2BP2, MET, GOT1, CARS1, SLC38A1, BBC3, AMY2B, ARHGAP9, NAMPT, IRF7, SHC1, MAP1LC3B, HERPUD1, CDC25B, PRELID3B, PTPN12, CCND2, CBX4, NFKBIZ, CXCL6, HS6ST2, SRXN1, ADM, GADD45A, SQSTM1, PHGDH, AARS1, INA, H1-0, RAB27A, ZNF274, POU5F1, AIFM2, SELL, MDM2, EIF2S2, OSGIN1, LINC00623, ITGB3, HSPA9, IL1B, NEFH, CDK13, STC2, ATF5, CTH, USP18, H2BC21, C3, DDIT3, SLC1A4, DDR2, PSAT1, SOD3, ZFAND2A, ABCA1, LAMP3, HSPA5, TNFSF13B, PLIN2, ENOSF1, GCLM, IGFBP4, TREM1, SLC2A3, TRIB1, EPSTI1, SEPHS2, FGF2, PTX3, SLIT2, ODF3B, IFRD2, TRIM16L, VEGFA, THRB, BCAT1, ASS1, SLC7A11, AREG, CASP4, EDNRA, TTC4, TES, MYC, JADE2, LINC02806, NR0B1, NRG1, TXNRD1, SLC30A1, TSC22D3, ARHGEF2, CASP8, RAB11FIP2, RASSF5, SESN2, WNT7B, SHB, ETS1, SLC3A2, ASNS, DENND2D, SNHG8, NFIL3, ATF4, MAFF, LY96, IFRD1, TNFRSF10B, PAPPA, IKBKE, PRNP, FOSL1, NNMT, MT1M, MMP1, GGT5, MAL2, EDEM1, SCN9A, HDAC9, INHBE, CHAC1, NMRAL2P, XBP1, AHR, NFKBIB, PVR, SYTL1, EEF1A2*
Overlapping DEGs between TNFα and combined treatment with TNFα+IFNγ	39	*L1CAM, BID, VCAM1, RELB, SOD2, TNFAIP2, NFKBIE, CXCL5, IRAK2, COL6A3, CCN4, CXCL2, CLDN1, NFKB1, TNFRSF9, DUSP4, KITLG, NFKBIA, TNFSF10, NFKB2, NFE2L3, CEBPB, GJB2, ICAM1, TNFRSF1B, PCDH8, CXCL1, TNFAIP3, STAP2, CCL2, TRAF1, TPBG, CXCL8, COL16A1, TNIP1, HMGA2, BIRC3, MMP9, STEAP1*
Overlapping DEGs between IFNγ and combined treatment with TNFα+IFNγ	48	*ERC5, HLA-F, IDO1, RSAD2, WARS1, HLA-DMA, HLA-DRB1, HLA-DRB4, IFITM3, HLA-DRB5, NRP1, TRIM22, DDX60, CMPK2, SERPINE2, SP110, IFI6, HERC6, LAP3, SAMHD1, NLRC5, H2AC6, HLA-E, IL15, IFI44L, CSF1, DTX3L, GBP1, CD74, PLSCR1, HLA-DRA, MDK, PSME2, IFITM1, CXCL9, TAP1, ISG20, BTN3A3, TXNIP, UBE2L6, STAT1, HLA-DQA1, HLA-DQB1, IFI44, PARP14, ISG15, IFIT2, IFITM2*
Overlapping DEGs between TNFα, IFNγ and combined treatment with TNFα+IFNγ	30	*C1S, C1R, CTSS, OAS1, MX1, IRF1, PLAAT4, HLA-B, SAMD9L, HLA-A, CFB, CASP1, APOBEC3G, XAF1, B2M, CFHR1, BTN3A2, HLA-C, GBP5, ACSL5, IFI30, PSMB8, MT2A, CFH, CXCL11, TAPBP, PSME1, PSMB9, IFIT3, IFI27*

Table S4. Overview and exact significance numbers of top 20 deregulated transcription factors (72 h) [1]

		p-values (Log10)		
**Gene set**	**Description**	**IFNγ**	**TNFα**	**TNFα+IFNγ**
TRR01275	Regulated by: STAT1	-8,9	-8,3	-16,7
TRR01277	Regulated by: STAT3	-5,9	-8,0	-16,3
TRR00602	Regulated by: IRF1	-7,5	-10,0	-10,2
TRR00875	Regulated by: NFKB1	-8,7	-24,3	-34,0
TRR01158	Regulated by: RELA	-8,8	-24,5	-34,3
TRR00117	Regulated by: CIITA	-28,0	-7,5	-17,5
TRR00489	Regulated by: HIVEP2	-11,6	-6,5	-9,4
TRR01167	Regulated by: RFXAP	-21,0	0,0	-10,3
TRR01166	Regulated by: RFXANK	-21,0	0,0	-10,3
TRR01165	Regulated by: RFX5	-19,9	0,0	-9,6
TRR00645	Regulated by: JUN	0,0	-7,8	-17,0
TRR01256	Regulated by: SP1	0,0	-6,0	-13,0
TRR00877	Regulated by: NFKBIA	0,0	-10,9	-11,1
TRR01157	Regulated by: REL	0,0	-8,2	-6,9
TRR01538	Regulated by: XBP1	0,0	-4,7	-9,3
TRR00466	Regulated by: HDAC1	0,0	-5,7	-10,9
TRR00025	Regulated by: ATF4	0,0	-4,0	-21,2
TRR01419	Regulated by: TP53	0,0	0,0	-11,1
TRR00075	Regulated by: BRCA1	0,0	0,0	-11,2
TRR01062	Regulated by: PPARG	0,0	0,0	-9,9

Table S5. Overview and exact significance numbers of top 20 enriched gene sets (72 h) [1].

		p-values (Log10)		
**Pathway**	**Description**	**IFNy**	**TNFα**	**TNFα+IFNy**
WP254	Apoptosis	0,0	-13,7	-20,4
R-HSA-8953897	Cellular responses to stimuli	0,0	-6,2	-23,2
GO:0097190	apoptotic signaling pathway	0,0	-8,5	-17,8
WP5087	Malignant pleural mesothelioma	0,0	-7,9	-16,1
WP4754	IL-18 signaling pathway	0,0	-24,5	-23,5
WP4925	Unfolded protein response	0,0	0,0	-17,9
R-HSA-449147	Signaling by Interleukins	-5,4	-18,6	-22,0
GO:0009617	response to bacterium	-6,6	-19,4	-26,6
WP5088	Prostaglandin signaling	-3,7	-9,6	-17,9
GO:0002521	leukocyte differentiation	-6,5	-7,8	-14,3
GO:0016032	viral process	-14,4	-8,0	-17,4
WP619	Type II interferon signaling (IFNG)	-20,7	-7,4	-17,1
GO:0050778	positive regulation of immune response	-23,9	-5,6	-15,3
R-HSA-1280218	Adaptive Immune System	-26,8	-11,1	-19,9
ko04612	Antigen processing and presentation	-38,6	-10,6	-24,7
R-HSA-1280215	Cytokine Signaling in Immune system	-47,9	-37,0	-58,8
GO:0031347	regulation of defense response	-18,0	-11,3	-21,3
GO:0019221	cytokine-mediated signaling pathway	-13,4	-15,7	-25,8
WP5115	Network map of SARS-CoV-2 signaling pathway	-16,3	-20,0	-26,7
GO:0001817	regulation of cytokine production	-15,6	-10,8	-16,7

Table S6. Overview of primers used for real-time qPCR.

Target	Sequence (For)	Sequence (Rev)
GFAP	CGAGATCGCCACCTACAGGAAGC	CCTTAATGACCTCTCCATCCCGC
S100B	CTTAGAGGAAATCAAAGAGCAGGAGGT	CATGTTCAAAGAACTCGTGGCAGG
SOX9	AGGAAGCTCGCGGACCAGTA	CTGCCCGTTCTTCACCGACT
MAO-B	CTCTGGGGGCTGCTACACAAC	CAGGGACATCCACAGACTCTGG
NFIA	CCCAGCACATCCTCTACGAG	AGGGGCTGCTGAAACCAGAC
AQP4	AACCCTGCAGTGACTGTGGC	CAGGAGACCATGACCAGCGG
CD44	AGCATCGGATTTGAGACCTG	GTTGTTTGCTGCACAGATGG
A2M	GAGGCGGAGAATGACGTACT	TCTTGGGTTGGTCCTTTCAC
PGK1	GATGGGCTTGGACTGTGGTC	ACAGCAAGTGGCAGTGTCTCC
RPL13A	GGTATGCTGCCCCACAAAACC	CTGTCACTGCCTGGTACTTCCA

Table S7. Antibodies used for immunofluorescent staining.

Target	Species	Dilution	Supplier	Catalogue number
		**Primary**		
AQP4	Rabbit	1:200	Santa Cruz	sc-20812
NESTIN	Mouse IgG1	1:200	R&D Systems	MAB1259
CD44	Rat	1:200	eBioscience/Thermo	
GFAP	Chicken	1:1000	Merck Millipore	AB5541
MAO-B	Rabbit	1:200	Sigma	HPA002328
NFkB p65	Rabbit	1:200	BioLegend	901301
S100B	mouse IgG1	1:200	Sigma	S 2532
VIMENTIN	Rabbit	1:200	Abcam	Ab92547
		**Secondary**		
Goat anti-Rat IgG (H+L), Alexa Fluor™ 647		1:1000	Invitrogen	A-21247
Goat anti-Rat IgG (H+L), Alexa Fluor™ 555		1:1000	Invitrogen	A-21434
Goat anti-Rat IgG (H+L), Alexa Fluor™ 488		1:1000	Invitrogen	A-11006
Goat anti-Chicken IgY (H+L), Alexa Fluor™ 647		1:1000	Invitrogen	A-21449
Goat anti-Chicken IgY (H+L), Alexa Fluor™ 555		1:1000	Invitrogen	A-21437
Goat anti-Chicken IgY (H+L), Alexa Fluor™ 488		1:1000	Invitrogen	A-11039
Goat anti-Mouse IgG1, Alexa Fluor™ 647		1:1000	Invitrogen	A-21240
Goat anti-Mouse IgG1, Alexa Fluor™ 555		1:1000	Invitrogen	A-21127
Goat anti-Mouse IgG1, Alexa Fluor™ 488		1:1000	Invitrogen	A-21121
Donkey anti-Rabbit IgG (H+L), Alexa Fluor™ 647		1:1000	Invitrogen	A-31573
Goat anti-Rabbit IgG (H+L), Alexa Fluor™ 555		1:1000	Invitrogen	A-21428
Chicken anti-Rabbit IgG (H+L), Alexa Fluor™ 488		1:1000	Invitrogen	A-21441

Table S8. Antibodies used for western blot.

Target	Species	Dilution	Supplier	Catalogue number
**Primary**				
P-STAT3 (Tyr705)	Rabbit	1:1000	Cell signaling	9145P
P-STAT1 (Tyr701)	Rabbit	1:1000	Cell signaling	7649S
GAPDH	Mouse	1:5000	Abcam	Ab8245
**Secondary**				
Mouse	Goat anti-mouse HRP antibody	1:2500	Jackson Immuno Research	115-035-174
Rabbit	Donkey anti-rabbit HRP antibody	1:5000	GE Healthcare	NA934v

References

1. Zhou, Y., Zhou, B., Pache, L., Chang, M., Khodabakhshi, A. H., Tanaseichuk, O., Benner, C. and Chanda, S. K. Metascape provides a biologist-oriented resource for the analysis of systems-level datasets. *Nat Commun* **2019**, *10,* 1523 doi: 10.1038/s41467-019-09234-6.
2. Reinhardt, P., Glatza, M., Hemmer, K., Tsytsyura, Y., Thiel, C. S., Hoing, S., Moritz, S., Parga, J. A., Wagner, L., Bruder, J. M., Wu, G., Schmid, B., Ropke, A., Klingauf, J., Schwamborn, J. C., Gasser, T., Scholer, H. R. and Sterneckert, J. Derivation and expansion using only small molecules of human neural progenitors for neurodegenerative disease modeling. *PLoS One* **2013**, *8,* e59252 doi: 10.1371/journal.pone.0059252.
3. Klima, S., Brull, M., Spreng, A. S., Suciu, I., Falt, T., Schwamborn, J. C., Waldmann, T., Karreman, C. and Leist, M. A human stem cell-derived test system for agents modifying neuronal N-methyl-D-aspartate-type glutamate receptor Ca(2+)-signalling. *Arch Toxicol* **2021**, *95,* 1703-1722 doi: 10.1007/s00204-021-03024-0.
4. Labib, D., Wang, Z., Prakash, P., Zimmer, M., Smith, M. D., Frazel, P. W., Barbar, L., Sapar, M. L., Calabresi, P. A., Peng, J., Liddelow, S. A. and Fossati, V. Proteomic Alterations and Novel Markers of Neurotoxic Reactive Astrocytes in Human Induced Pluripotent Stem Cell Models. *Front Mol Neurosci* **2022**, *15,* 870085 doi: 10.3389/fnmol.2022.870085.
